# Kinetic Reaction Mechanism of Sinapic Acid Scavenging NO_2_ and OH Radicals: A Theoretical Study

**DOI:** 10.1371/journal.pone.0162729

**Published:** 2016-09-13

**Authors:** Yang Lu, AiHua Wang, Peng Shi, Hui Zhang, ZeSheng Li

**Affiliations:** 1 College of Material Science and Engineering, Harbin University of Science and Technology, Harbin, People’s Republic of China; 2 College of Chemical and Environmental Engineering, Harbin University of Science and Technology, Harbin, People’s Republic of China; 3 Key Laboratory of Cluster Science of Ministry of Education & School of Chemistry, Beijing Institute of Technology, Beijing, People’s Republic of China; University of Calgary, CANADA

## Abstract

The mechanism and kinetics underlying reactions between the naturally-occurring antioxidant sinapic acid (SA) and the very damaging ·NO_2_ and ·OH were investigated through the density functional theory (DFT). Two most possible reaction mechanisms were studied: hydrogen atom transfer (HAT) and radical adduct formation (RAF). Different reaction channels of neutral and anionic sinapic acid (SA^-^) scavenging radicals in both atmosphere and water medium were traced independently, and the thermodynamic and kinetic parameters were calculated. We find the most active site of SA/SA^-^ scavenging ·NO_2_ and ·OH is the –OH group in benzene ring by HAT mechanism, while the RAF mechanism for SA/SA^-^ scavenging ·NO_2_ seems thermodynamically unfavorable. In water phase, at 298 K, the total rate constants of SA eliminating ·NO_2_ and ·OH are 1.30×10^8^ and 9.20×10^9^ M^-1^ S^-1^ respectively, indicating that sinapic acid is an efficient scavenger for both ·NO_2_ and ·OH.

## Introduction

Sinapic acid (SA, 3,5-dimethoxy-4-hydroxycinnamic acid) is a naturally-occurring and widespread phenolic acid in the plant kingdom, and can be obtained from various fruits and vegetables such as rye [1], orange, grapefruit, and cranberry [2]. Especially, SA accounts for over 73% of all free phenolic acids in rapeseed [3]. SA is a bio-active compound reported as anti-inflammatory and anxiolytic ingredient [4,5]. In addition, SA is a widely-investigated antioxidant, and because of the peroxynitrite (ONOO^-^) scavenging activity [6], it can be utilized to promote the cellular defense activity against the diseases involving ONOO^-^ [7].

Hydroxycinnamic acid is a particularly important group of phenolic acids, mainly including caffeic acid, ferulic acid, *p*-coumaric acid, and sinapic acid, which exist in daily foods such as grape, citrus [8], pear, tomato, and small radish [2]. Antioxidant activities of hydroxycinnamic acids have been studied extensively [9–12]. Among, SA is suggested as a more efficient antioxidant [13] and superoxide radical scavenger [14] than *p*-coumaric acid and ferulic acid. Cuvelier et al. have also confirmed that, in a lipophilic solvent, the antioxidant activity of SA is higher than that of *p*-coumaric acid, ferulic acid, and syringic acid [15].

Owing to the high radical scavenging ability, SA is a good protector against the oxidative damage caused by the accumulation of redundant free radicals in vivo, which are the primary cause of many chronic diseases [16–20]. Thus, it is vital to analyze the viable mechanisms underlying how SA eliminates different radicals. Despite having some experimental research, there are only three theoretical studies about the antioxidant capacity of SA to our best knowledge. First, Bakalbassis et al. investigated the structure-antioxidation relationships of SA and other three hydroxycinnamic acid derivatives using ab initio and DFT methods. By calculating the absolute infrared intensity, heat of formation, and electron-donating ability, they evaluated the antioxidant activities of SA and other derivatives, and found that both degree of conjugation and extent of spin delocalization in phenoxyl radicals affect the scavenging activity of phenolic acid [21]. Second, Galano et al. theoretically studied the ·OOH scavenging activity of SA in aqueous and lipid solutions using DFT. They predicted the rate coefficient of SA with ·OOH, and concluded SA is a good ·OOH scavenger [22]. Third, Urbaniak et al. studied the antioxidant properties of *p*-coumaric acid and SA, and obtained the bond dissociation enthalpy, adiabatic ionization potential, proton dissociation enthalpy, proton affinity, electron transfer enthalpy, gas phase acidity, and Gibbs energy at the DFT/B3LYP. They confirmed the high antioxidant activities of both compounds, and indicated O-H is the preferred place of free radical attack [23].

It can be seen the theoretical research concerning the antioxidant activity of SA are really little. Even for the theoretical research of phenolic acids in the same area, the focus are almost on the structural and electronic properties of antioxidants, such as bond dissociation energy (BDE), adiabatic ionization potential (AIP), and spin density (SD) etc [23–30]. The reaction mechanisms of phenolic acids with specific radicals are rarely investigated. Moreover, reactive oxygen species (ROS) attracted more attention [21–22, 31–33], while another main byproducts in human body: reactive nitrogen species (RNS) are unnoticed. Thus, the main goal of this work is to carry out a systematical theory study on the reaction mechanisms of SA with two important RNS and ROS (·NO_2_ and ·OH), and provide the thermodynamic and kinetic details of all possible reaction channels. Hope our results are helpful for designing the high-activity scavengers against RNS and ROS.

·NO_2_ and ·OH were chosen because they are the typical radicals of RNS and ROS, respectively, in biosystems. Excessive production of RNS and ROS may exert pathological stress to cells and tissues. This oxidative stress can weaken the defense system and render the tissues more sensitive to the subsequent insult. Thus, such mechanism can cause cell damages including oxidative damage to essential proteins, lipid peroxidantion, and DNA strand breakage. As a common RNS, ·NO_2_ reportedly can induce lipid peroxidation, membrane damage and cell death [34–36]. And in aqueous solution, ·NO_2_ could be hydrolyzed to ONOO^-^, NOO^-^ or other like harmful species, so it is significant to investigate the ·NO_2_ eliminating mechanism. As for ·OH, it is the most active and damaging ROS in biosystems, with a very short half-life of about 10^−9^ s [37]. Thus, the study of kinetic mechanisms how SA scavenges these two radicals are valuable to the further design of phenolic antioxidants.

It is generally assumed that the reactions between phenolic compounds and radicals (·R) proceeds through three main mechanisms: direct H-transfer process from the antioxidant molecule (Eq 1), radical adduct formation (Eq 2) and single-electron transfer process (Eqs 3 and 4):

Hydrogen atom transfer (HAT):

SA+⋅R→⋅SA(-H)+RH (1)

Radical adduct formation (RAF):

SA+⋅R→⋅(SA−R) (2)

Single-electron transfer (SET):

$$\text{SA}+\cdot \text{R}\to \cdot {\text{SA(-H)}}^{+}+{\text{RH}}^{\text{-}}$$ (3)

$$\cdot {\text{SA(-H)}}^{+}+{\text{RH}}^{\text{-}}\to \cdot \text{SA(-H)}+\text{RH}$$ (4)

The previous theoretical studies on the homologous phenolic compounds such as gallic acid, caffeic acid, and sinapic acid have shown that SET mechanism dose not play a relevant role in the reactions with free radicals [31, 38–40]. Hence, in the present work, we are focused on HAT and RAF as the most probable reaction mechanisms.

The contribution of two mechanisms were analyzed. All possible reaction/attack sites were examined, and the corresponding channels were identified by thermodynamic and kinetic calculations. Rate constants and branching ratios for different channels were also estimated. To simulate the water-dominated cellular environment, we also took into account the effect of aqueous solution. Then the theoretical and experimental results were compared.

## Computational Methods

DFT calculations were carried out using the GAUSSIAN 09 computational package [41]. Geometry optimization and frequency calculations were performed at the M05-2X level [42] with the basis set 6–311++G(d,p). The M05-2X functional was recommended for kinetic calculation by the developers [43] and was successfully used by independent authors with that purpose [44–51]. It is also one of the best functional for calculating the reaction energy involving free radicals [52].

Unrestricted calculations were used for open shell systems. The nature of stationary points was evaluated by using normal vibration frequencies: all of reactants (R), complexes (C) and products (P) must show positive real frequencies; all of transition states (TSs) must show a single imaginary frequency which corresponds to the expected vibration mode. In addition, the intrinsic reaction coordinates (IRC) at the M05-2X/6-311++G(d,p) level were calculated to obtain the minimum energy path.

Solvent effects were introduced using the continuum solvation model based on solute electron density (SMD) [53], which is a universal solvation model due to its applicability to either charged or uncharged solute in any solvent or liquid medium. At the same level of M05-2X/6-311++G(d,p), all reactants and products were optimized in aqueous solution by SMD model. The transition states were also optimized using SMD model in aqueous solution. However, in spite of trying our best, only part of the TS for water-phase pathways were identified. Therefore, the solvation effect on TS were estimation by the single point calculations with SMD model on the basis of the optimized gas-phase geometries. Then, we compared the geometries of TS found in aqueous solution with the corresponding TS optimized in gas phase (see S1 Fig). The results show that the geometries of TS optimized in aqueous solution are very similar with that of TS optimized in the gas phase. Such as, for the bond of readying to form, the biggest difference in bond length is only 0.13Å. We also compared the energy barrier heights obtained by optimization of TS under SMD model with that of obtained by the single point calculation using SMD model based on the gas-phase optimized geometries of TS (see S1 Table). It can be seen that the differences in energy barrier height are also little. Hence, we think that the single energy calculation using SMD model seems to provide a proper estimation of solvent effects for our studied systems. Just as reported in the literature [54–56], the results of single point calculations using solvent model were in good agreements with the corresponding experimental results for their systems.

Reaction enthalpy in solution was computed by the difference of enthalpy values between products and reactants optimized in the presence of SMD model. Relative Gibbs energy in solutions was computed using thermodynamic cycle and Hess’ law which explicitly include solvation energy. For example, the thermodynamic cycle for the addition reaction between SA and ·OH is as follows:

$$\begin{array}{l} {\text{SA}}_{\text{gas}}+\cdot {\text{OH}}_{\text{gas}}\overset{\Delta G_{\text{gas}}}{\to }\cdot {\text{(SA-OH)}}_{\text{gas}}\\ {↑}^{-\Delta G\text{s}\text{(SA)}}{↑}^{-\Delta G\text{s}\text{(}\cdot \text{OH)}}{↓}^{\Delta G\text{s}\cdot \text{(SA-OH)}}\;\\ {\text{SA}}_{\text{sol}}+\cdot {\text{OH}}_{\text{sol}}\overset{\Delta G_{\text{sol}}}{\to }\cdot {\text{(SA-OH)}}_{\text{sol}} \end{array}$$ (5)

With this strategy, the Gibbs energy of reaction in solution (Δ*G*_sol_) can be determined as the sum of the Gibbs energy of reaction in the gas phase (Δ*G*_gas_) and the difference in solvation energies (ΔΔ*G*_s_):

$$\Delta G_{\text{sol}}=\Delta G_{\text{gas}}^{}+\Delta \Delta G_{\text{s}}$$ (6)

where ΔΔ*G*_s_ is calculated as:

$$\Delta \Delta G_{\text{s}}=\Delta G_{\text{s}}\cdot \text{(SA}-\text{OH)}-\Delta G_{\text{s}}\text{(SA)}-\Delta G_{\text{s}}\text{(}\cdot \text{OH)}$$ (7)

where Δ*G*_s_ is the solvation energy. The reference state is 1M in all cases. The solvent cage effect was included with Okuno’s corrections [57], which take into account the free volume theory. These corrections agree well with those independently obtained by Ardura et al. [58]. In this work the expression used to correct the Gibbs energy as follows:

$$\Delta G_{\text{sol}}^{\text{FV}}≅\;\Delta G_{\text{sol}}^{\text{0}}-RT\left\{\text{ ln }\left[n10^{\left(2n-2\right)}\right]-\text{(}n-1\text{)}\right\}$$ (8)

where *n* is the reaction molecularity. According to Eq 8, the solvent cage effect causes a decrease of 10.63 kJ/mol in Δ*G* for a bi-molecular reaction at 298.15 K [59].

The theoretical rate constants of all channels were calculated using the theory of improved canonical variational transition state (ICVT) [60] with small-curvature tunneling (SCT) correction [61] on the program POLYRATE 9.7 [62]. We adopted a separable equilibrium solvation (SES) approximation [63] to calculate the rate constants of water-phase reactions. Specifically, we first calculated the gas-phase reaction channels, solved each configuration along the gas-phase IRC (including reactants, products and saddle points), and then calculated the water-phase rate constants through the variational transition state theory with interpolated single-point energy (VTST-ISPE) on POLYRATE.

## Results and Discussion

**Fig 1:**
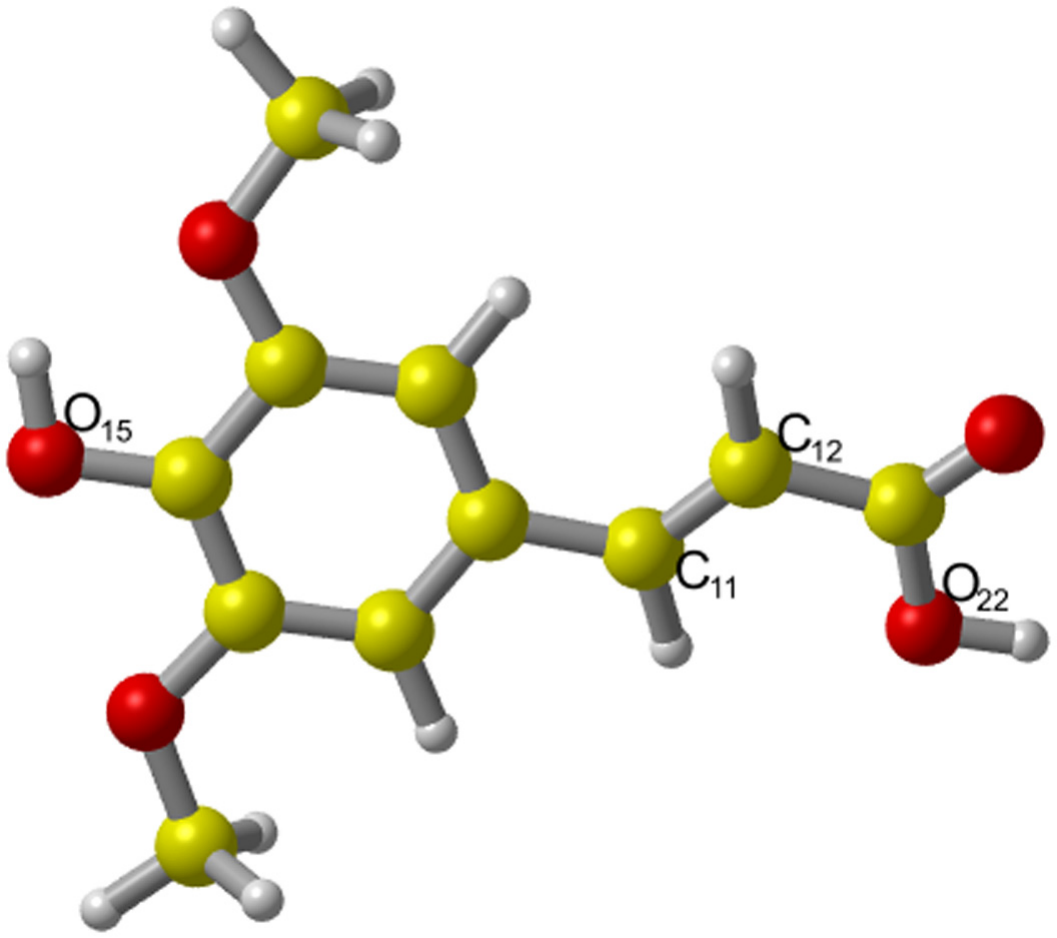
The optimized geometry of SA in the gas phase. The result show that SA has an approximately planar structure. The dihedral angle between benzene ring and carbonyl group is ~179.56.

The optimized structure of SA as well as the atomic numbering scheme are shown in Fig 1. Clearly, SA has an approximately planar structure in which the dihedral angle between benzene ring and carbonyl group is about 179.56°. This result is consistent with the reported structure of SA optimized under the B3LYP/6-31+G(d) level [64]. The planar structure of SA implies that the molecule is completely conjugated and lead to an extended spin delocalization [65]. The possibility of excellent delocalization could account for its potential radical scavenging activity [65].

### ·NO_2_ scavenging by SA

For reactions of SA with ·NO_2_, we considered two most possible HAT channels from -OH and -COOH, and defined them as channels O_15_ and O_22,_ respectively (on sites O_15_ and O_22_ in Fig 1); we also considered two RAF channels with addition of the corresponding radical to the carbon-carbon double bond, and defined them as channels C_11_ and C_12_, respectively (on sites C_11_ and C_12_ in Fig 1).

**Fig 2:**
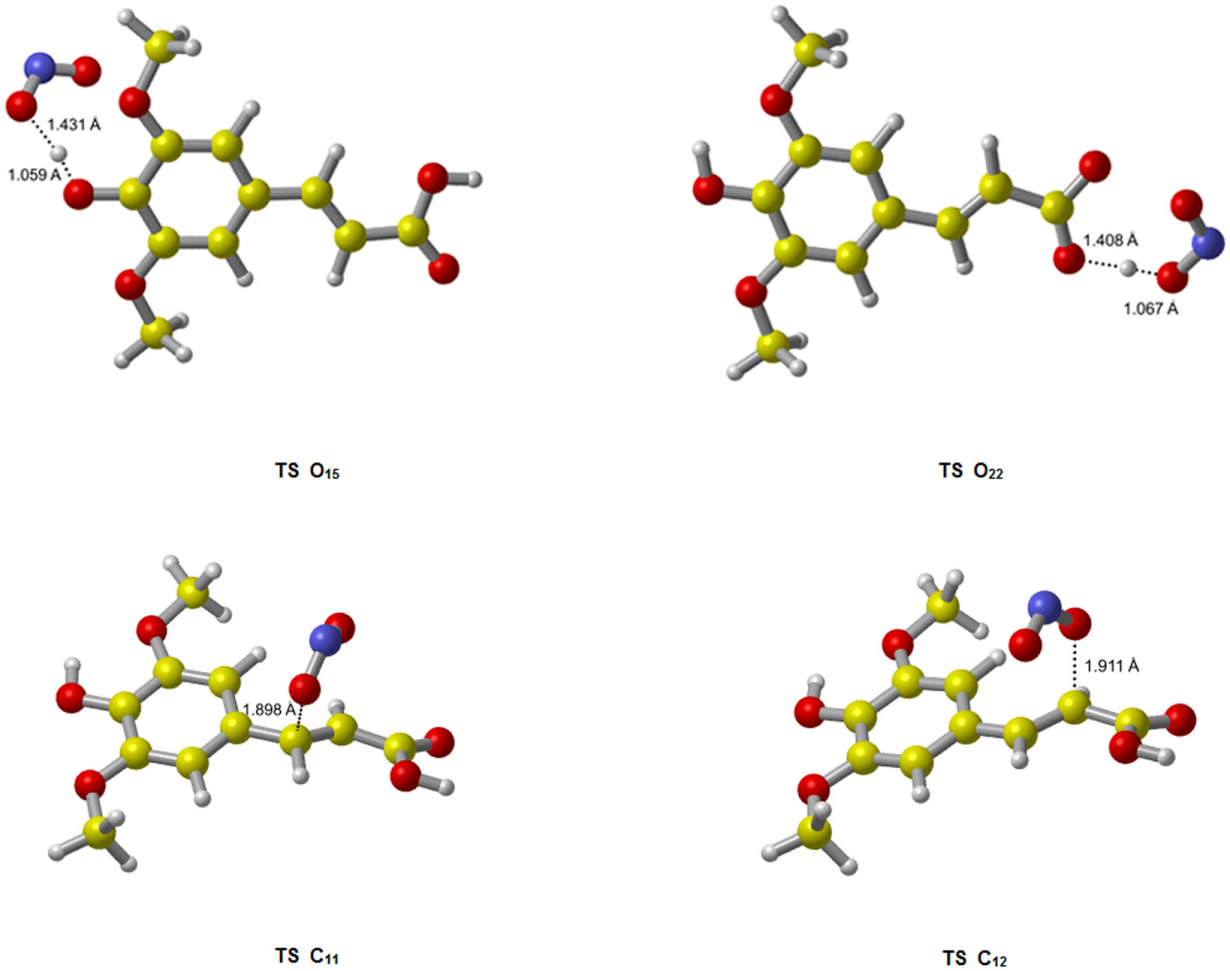
The transition state geometries for the reactions of SA with ·NO_2_. Channel O_15_ has an earlier TS relative to channel O_22_, associated with a lower reaction energy barrier. This agree with our calculation results in Table 1.

**Fig 3:**
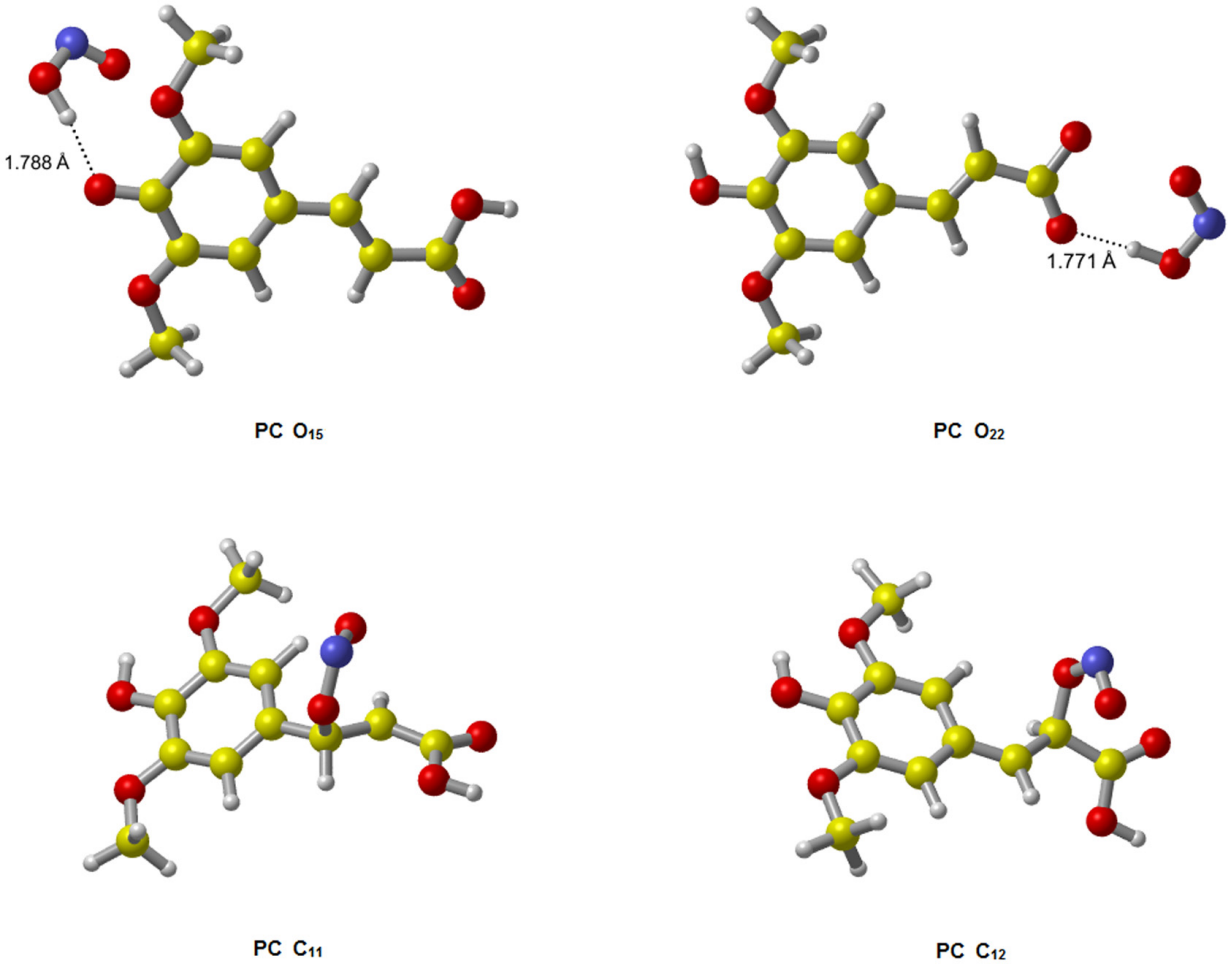
The optimized geometries of product complexes for the reactions of SA with ·NO_2_. In HAT product complexes structures, HONO molecules form hydrogen bonds with SA semiquinone radicals.

The optimized geometries of TSs and product complexes (PCs) of all channels are shown in Figs 2 and 3. The reaction enthalpy (Δ*H*), Δ*G* and energy barrier height (Δ*E*) including zero-point energy (ZPE) corrections obtained for each channel were collected in Table 1.

**Table 1:** The reaction enthalpies (Δ*H*), reaction Gibbs energies (Δ*G*) and energy barrier heights with ZPE corrections (Δ*E*+ZPE), at 298 K, for the reactions of SA with ·NO_2_ in the gas phase and water phase (in kJ/mol).

SA+·NO_2_	Δ*H*_gas_	Δ*H*_sol_	Δ*G*_gas_	Δ*G*_sol_	Δ*E*_gas_+ZPE	Δ*E*_sol_+ZPE
**O**_**15**_	19.09	-7.63	16.78	-25.42	42.58	-8.79
**O**_**22**_	142.85	57.59	136.15	-58.39	109.25	117.18
**C**_**11**_	26.76	11.70	77.34	51.90	57.39	44.84
**C**_**12**_	-0.63	-12.57	51.82	29.61	50.29	32.15

According to the values of Δ*G* (Table 1), only HAT channels in water phase are exergonic (Δ*G*<0), meaning they could spontaneously occur. On the contrary, the RAF channels are all endergonic (Δ*G>*0) wherever in gas or water phase. Such, we conclude that the RAF mechanism is not thermodynamically feasible for SA scavenging ·NO_2_.

**Fig 4:**
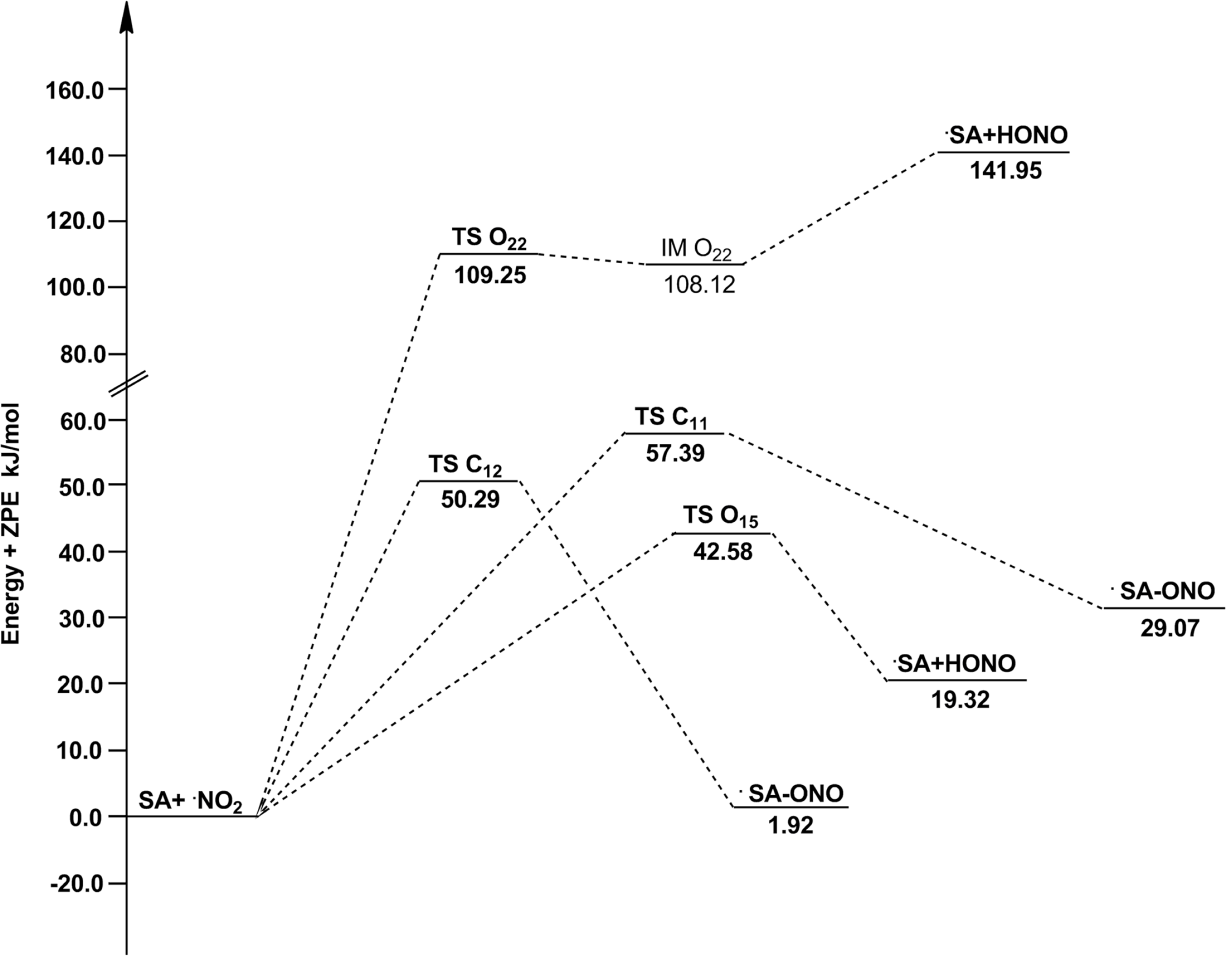
The potential energy surfaces for the reactions of SA with ·NO_2_ in the gas phase. The relative energies (in kJ/mol) were calculated at the M05-2X/6-311++G(d,p) + ZPE level. To facilitate the comparison, the energy of the reactants are set to zero.

**Fig 5:**
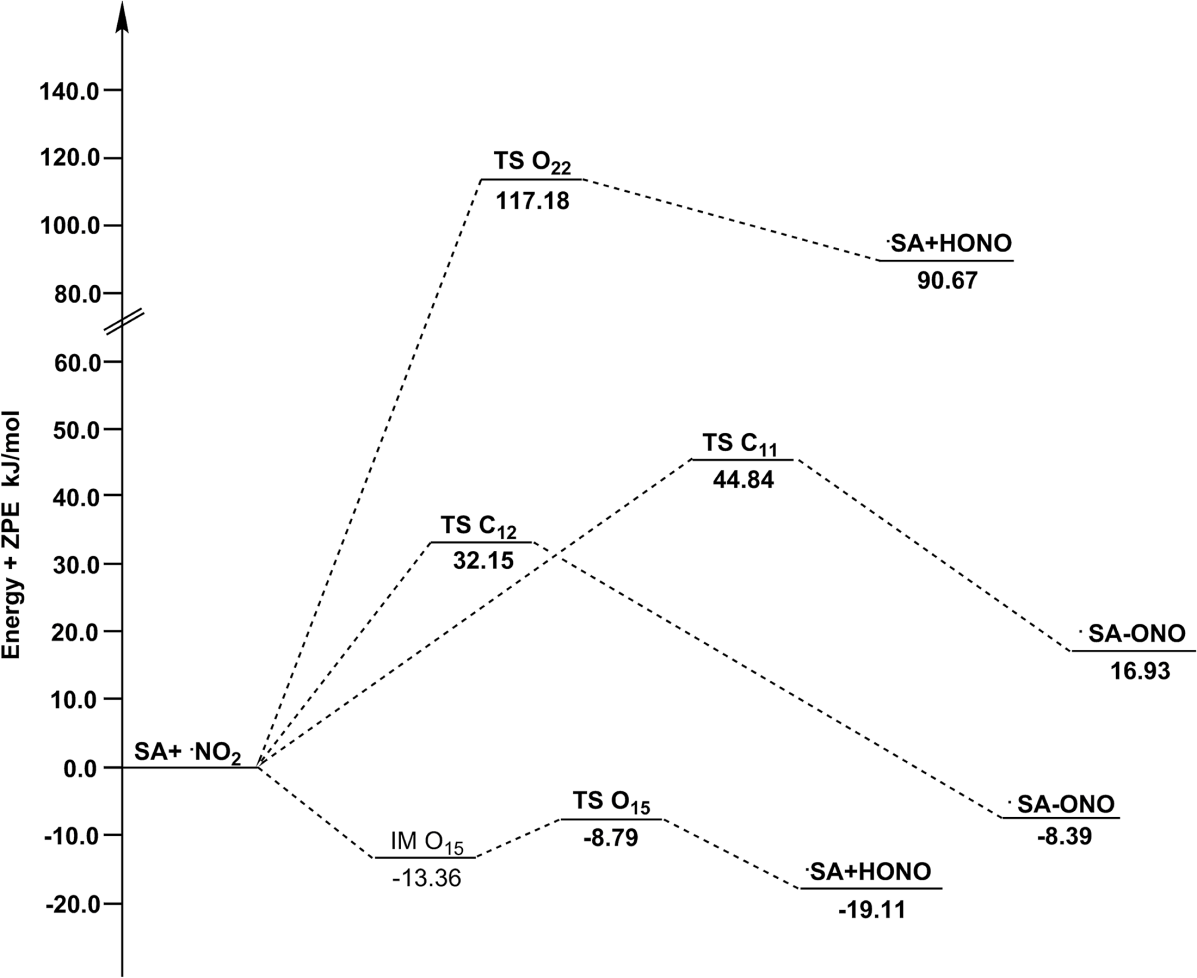
The potential energy surfaces for the reactions of SA with ·NO_2_ in water phase. The relative energies (in kJ/mol) were calculated at the M05-2X/6-311++G(d,p) + ZPE level. To facilitate the comparison, the energy of the reactants are set to zero.

As showed in Fig 2, within the HAT reactions, the TS O_15_ appears systematically earlier than the TS O_22_. Since the earlier TS is usually associated with lower reaction energy barrier, the site O_15_ should be more active. The results of Δ*E*+ZPE (Table 1) indeed show such a trend: channel O_15_ has lower energy barrier height than channel O_22_ both in gas and water phases. For RAF reactions, channel C_11_ and C_12_ have similar energy barrier heights and thus similar activities. The active order in terms of energy barrier height is as follows: O_15_ (42.58 kJ/mol) > C_11_ (50.29 kJ/mol) > C_12_ (57.39 kJ/mol) > O_22_ (109.25 kJ/mol) in gas phase, and O_15_ (-8.79 kJ/mol) > C_11_ (32.15 kJ/mol) > C_12_ (44.84 kJ/mol) >O_22_ (117.18 kJ/mol) in water phase. Thus, the medium does not seem to affect the order of reaction energy barrier or activity. In this case, channel O_15_ is the major channel for all reactions of SA with ·NO_2_. In addition, the water-phase channel O_15_ with negative Δ*E*+ZPE is a barrierless reaction, meaning a pre-reactive complex (IM) is formed in the entrance of the reaction, which energy is lower than that of the reactants, and the “real” energy barrier height between IM O_15_ and TS O_15_ is 4.57 kJ/mol. The details of relative energies are plotted in Figs 4 and 5.

The spin density is another important parameter to characterize the stability of free radicals formed from phenolic acids, because the energy of a free radical can be efficiently decreased if the unpaired electron is highly delocalized through the conjugated system [66]. The phenolic acid radical having higher spin density delocalization, easier is the formation of product and then higher radical-scavenging activity of the group. Atomic spin densities plots for the product radicals of all water-phase channels in S3 Fig indicate that when H-abstraction occurs on O_15_ atom, a broad delocalization of the unpaired electron involving the O_15_, almost all C atoms of benzene ring, C_11_ and C_12_ atoms occurs. While the RAF channels entail a small delocalization of the odd electron concentrating on the carbon-carbon double bond. The spin density of O_15_ atom (0.243) of Product O_15_ shows lager spin density concentration than O_22_ atom (0.016) of Product O_22_, which is further confirmed that O_15_H-group is more sensitive to the free-radical attack.

The rate constant and branching ratio of each channel, as well as the total rate constants at 298 K in both gas and water phases, were calculated out and listed in Table 2. We assumed that neither mixing nor crossover between different channels occurs and therefore calculated the total rate constants of HAT and RAF reactions for SA scavenging ·NO_2_ as follows:

$$k_{{\text{total-NO}}_{\text{2}}}\text{=}k_{{\text{HAT-NO}}_{\text{2}}}\;+\;k_{{\text{RAF-NO}}_{\text{2}}}$$ (9)

**Table 2:** The calculated ICVT/SCT rate constant (*k*) and branching ratios (Γ) for SA scavenging ·NO_2_, at 298 K, in the gas phase and water phase, together with the available experimental value from [67].

SA+·NO_2_	atmosphere		water		experiment
	*k* (M^-1^ S^-1^)	Γ(%)	*k* (M^-1^ S^-1^)	Γ(%)	*k* (M^-1^ S^-1^)
**O**_**15**_	3.06X10^5^	98.92	1.28X10^8^	99.99	7.2X10^8^
**O**_**22**_	3.18X10^3^	1.03	3.58X10^3^	0.01	
**C**_**11**_	4.94X10^1^	0.02	9.51X10^1^	~0	
**C**_**12**_	1.02X10^2^	0.03	1.58X10^2^	~0	
**Total**	3.1X10^5^		1.3X10^8^		

The rate constant of each mechanism was estimated by summing up the rate constants of different channels:

$$k_{{\text{HAT-NO}}_{\text{2}}}\text{=}k_{{\text{O}}_{\text{15}}}\;+\;k_{{\text{O}}_{\text{22}}}$$ (10)

$$k_{{\text{RAF-NO}}_{\text{2}}}\;=\;k_{{\text{C}}_{\text{11}}}\;+\;k_{{\text{C}}_{\text{12}}}$$ (11)

As showed in Table 2, channel O_15_ possesses the largest rate constant which is several orders of magnitude higher than other channels. The RAF channels C_11_ and C_12_ have similar rate constants, but which are much lower than HAT channels. In addition, for the same channel, the *k* in water phase is always higher than in gas phase, which is particularly evident on the channel O_15_, indicating that aqueous solutions can enhance the ·NO_2_ scavenging activity of SA.

To quantify the contribution of each channel to the total reaction, we calculated the branching ratio (Γ), representing the percent of each channel in the total rate constant, as follows:

$$\Gamma_{\text{i}}=\frac{k_{\text{i}}}{k_{\text{tatol}}}\times \text{100}$$ (12)

Also in Table 2, channel O_15_ contributes more than 98% capacity of scavenging ·NO_2_, indicating it accounts for almost the whole activity of SA. And the rest channels are very rarely used. Summarily, channel O_15_, combined with its thermodynamic superiority, is the absolutely dominant pathway for SA scavenging ·NO_2_. As for the RAF channels, since their total branching ratio is very little and they are significantly endergonic, it is reasonable that the RAF mechanism is irrelevant for the ·NO_2_ scavenging activity of SA in gas or water phase.

**Fig 6:**
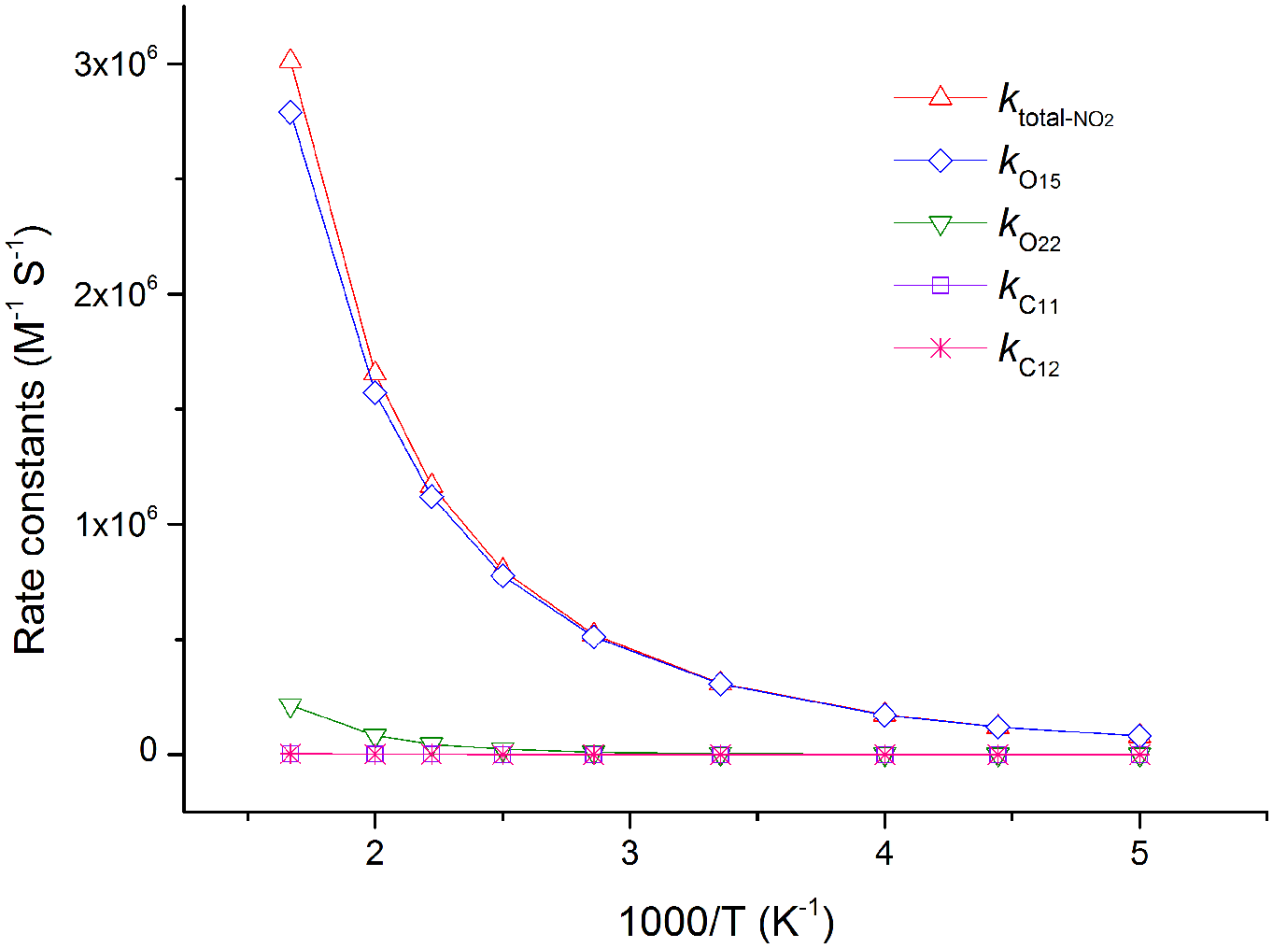
The calculated ICVT/SCT rate constants for the reactions of SA with ·NO_2_ and the total rate constants (*k*_total-NO2_) versus 1,000/T, from 200 K to 600 K, in the gas phase (in M^-1^ S^-1^). All of HAT and RAF channels have positive temperature dependence.

**Fig 7:**
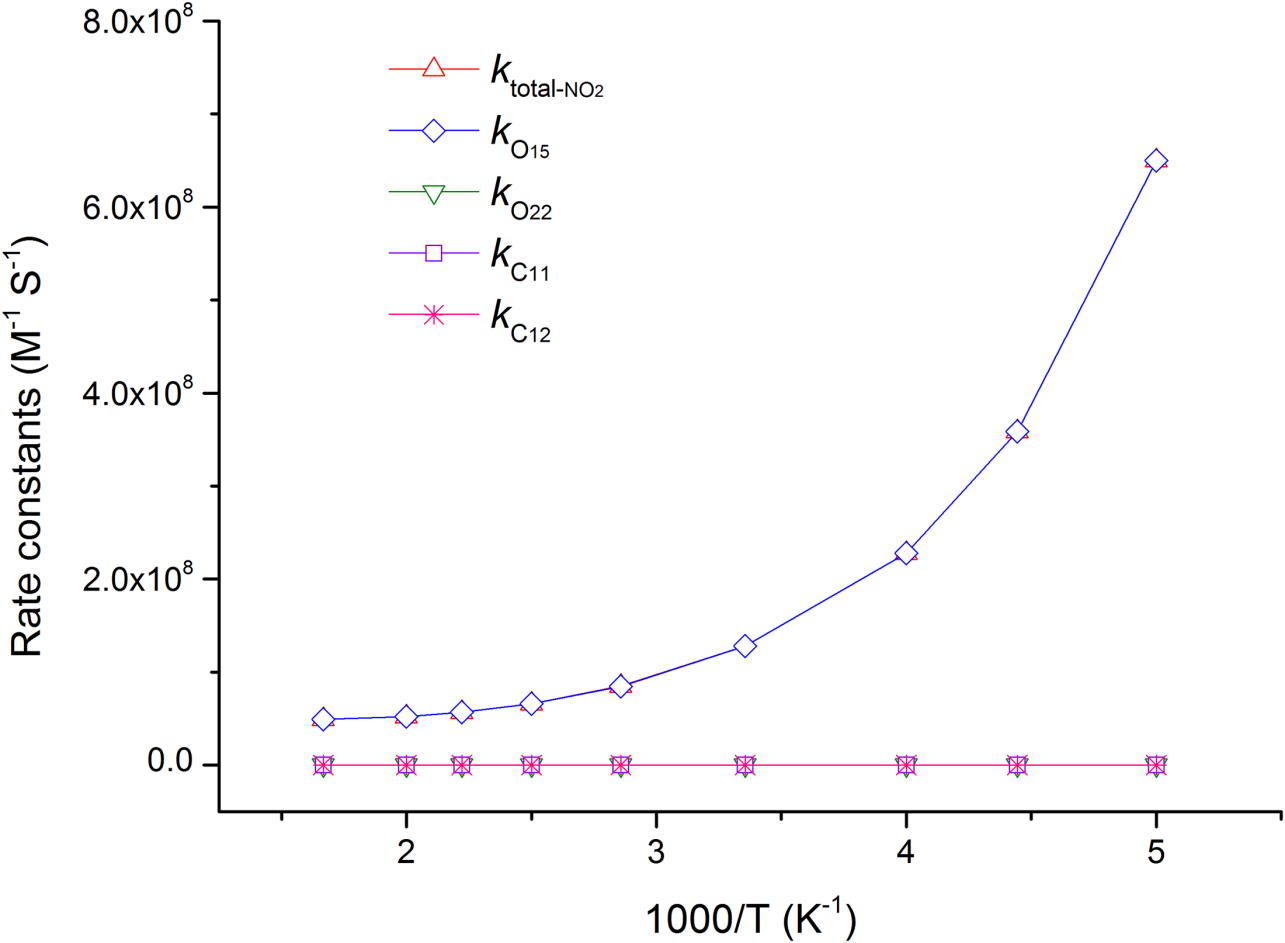
The calculated ICVT/SCT rate constants for the reactions of SA with ·NO_2_ and the total rate constants (*k*_total-NO2_) versus 1,000/T, from 200 K to 600 K, in water phase (in M^-1^ S^-1^). The HAT channel O_15_ has a negative temperature dependence, as a negative energy barrier within the reaction process; other channels have positive temperature dependence.

To better understand the kinetic mechanisms of the studied reactions, we calculated the temperature dependence of rate constant for each channel and plotted it against the reciprocal of temperature in Figs 6 and 7. Clearly, the HAT channel O_15_ is always keeping its superiority in the range of 200–600 K, which further confirms its leading role. The lower lines *k*_O22_, *k*_C11_ and *k*_C12_ are nearly overlapped, especially at lower temperature. The rate constant of channel O_15_ presents a negative temperature dependence, as its negative energy barrier (Fig 7).

The total rate constants at 298 K, as well as the experimentally- determined rate constant [67], are also listed in Table 2. In aqueous environment, the calculated total rate constant of SA scavenging ·NO_2_ is 1.30×10^8^ M^-1^ S^-1^, which is at the same order of magnitude as the experimental result (7.20×10^8^ M^-1^ S^-1^). This agreement validates the reliability of our calculations. Furthermore, it is important to compare the rate constant of SA scavenging ·NO_2_ with the rate constants of ·NO_2_ damaging unsaturated fatty acids in vivo. As reported, the rate constants in ·NO_2_-based oxidations of tyrosine, fumaric acid and linoleic acid are (M^-1^ S^-1^): *k* = 3.20×10^5^ [68], 1.30×10^7^ and 5.00×10^4^ [69], respectively, which are much smaller than the rate constant of SA eliminating ·NO_2_ predicted here. Thus, we conclude that SA is able to efficiently prevent cell damage by directly trapping ·NO_2_.

### ·OH scavenging by SA

Like the previous section, in order to investigate the antioxidant activity of SA toward ·OH, we identified two HAT channels O_15h_ and O_22h_, as well as two RAF channels C_11h_ and C_12h_. Because we concern the application of the antioxidant in vivo, and the obtained rate constants in aqueous solutions are closer to the fact in vivo environment, we only retained the thermodynamic and kinetic data of water phase in this section.

The results of Δ*H*, Δ*G* and Δ*E* including the ZPE corrections calculated under the level of M05-2X/6-311++g(d,p) are listed in Table 3. For scavenging ·OH, all HAT and RAF channels in aqueous solutions are exothermic and exergonic, indicating they are all thermodynamically feasible. Thus, SA is able to scavenge ·OH in vivo through both mechanisms of HAT and RAF. According to the spin densities plots in S3 Fig, similar with scavenging ·NO_2_, HAT channels have more extended delocalization of the unpaired electron, indicating that the HAT mechanism should be more efficient than the RAF mechanism.

**Table 3:** The reaction enthalpies (Δ*H*), reaction Gibbs energies (Δ*G*) and energy barrier heights with ZPE corrections (Δ*E*+ZPE), at 298 K, for the reactions of SA with ·OH in water phase (in kJ/mol).

SA+·OH	Δ*H*_sol_	Δ*G*_sol_	Δ*E*_sol_+ZPE
**O**_**15h**_	-169.11	-183.65	-54.14
**O**_**22h**_	-103.89	-216.61	-21.85
**C**_**11h**_	-126.86	-95.06	-3.20
**C**_**12h**_	-138.87	-104.48	-3.39

**Fig 8:**
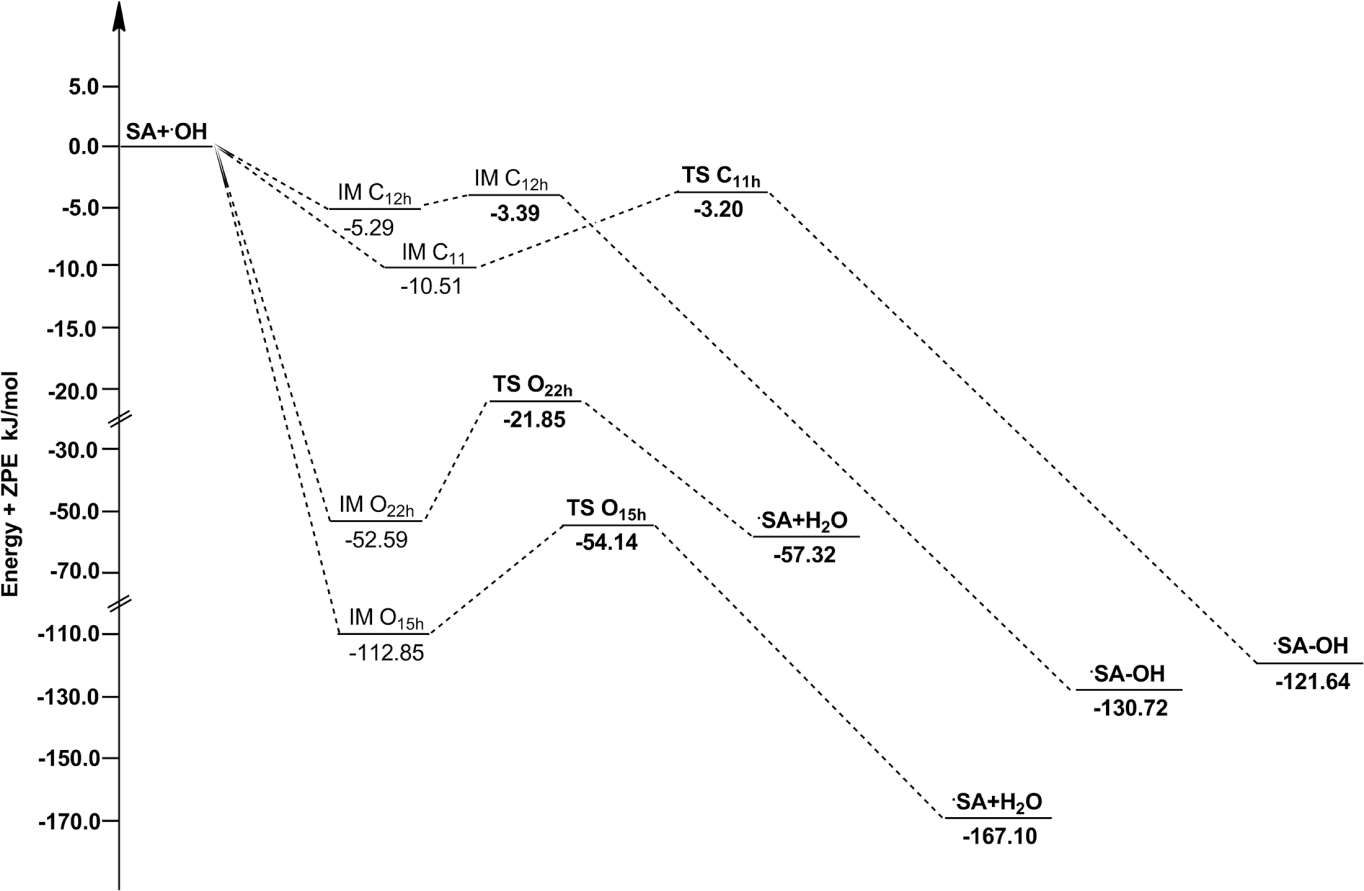
The potential energy surfaces for the reactions of SA with ·OH in water phase. The relative energies (in kJ/mol) were calculated at the M05-2X/6-311++G(d,p) + ZPE level. To facilitate the comparison, the energy of the reactants are set to zero.

As showed in Table 3, channel O_15h_ has a lower energy barrier height than channel O_22h_, it is the major channel of SA scavenging ·OH within the HAT mechanism. The -O_15_H group has higher activity mainly because the corresponding H-abstraction product is a more stable semiquinone radical, as the spin density of O_15h_ atom (0.243) of Product O_15h_ has lager spin density concentration than O_22h_ atom (0.016) of Product O_22h_. The activities of RAF channels are lower than HAT channels, and channels C_11h_ and C_12h_ are very little different in energy barrier heights. As mentioned before, the channels with negative energy barrier heights are barrierless reactions, and the IMs are formed in the entrances of the reactions. The “real” energy barrier heights need to overcome between IM O_15h_ and TS O_15h,_ IM O_22h_ and TS O_22h,_ IM C_11h_ and TS C_11h,_ IM C_12h_ and TS C_12h_ are 58.71, 30.74, 7.49 and 1.90 kJ/mol, respectively. The relative energies are plotted in Fig 8.

The rate constants and branching ratios of SA scavenging ·OH were also calculated and listed in Table 4, as well as the available experimental data. As showed in Table 4, the HAT channel O_15h_ possesses the largest rate constant, followed by the HAT channel O_22h_. The RAF channels C_11h_ and C_12h_ have similarly lower rate constants. Thus, to the total reaction of SA with ·OH, the most contributive pathway is channel O_15h_ (Γ_O15h_ = 62.97%). And different from the case of scavenging ·NO_2_ (Γ_O22_ = 0.01%), channel O_22h_ has a considerable contribution (Γ_O22h_ = 36.98%). The contributions of RAF channels (Γ_RAF_ = 0.05%) are estimated to be negligible. The total rate constants of HAT channels are on the 10^9^ at 298 K, meaning that the reactions are very fast and diffusion controlled, underlining the excellent antioxidant activity of SA toward ·OH.

**Table 4:** The calculated ICVT/SCT rate constant (*k*) and branching ratios (Γ) for SA scavenging ·OH, at 298 K, in water phase, together with the available experimental values (*k*_exp_) from [70].

site	*k* (M^-1^ S^-1^)	Γ(%)	*k*_exp_ (M^-1^ S^-1^)
**O**_**15h**_	5.79X10^9^	62.97	9.6X10^9^
**O**_**22h**_	3.40X10^9^	36.98	
**C**_**11h**_	3.46X10^6^	0.03	
**C**_**12h**_	2.30X10^6^	0.02	
**Total**	9.2X10^9^		

**Fig 9:**
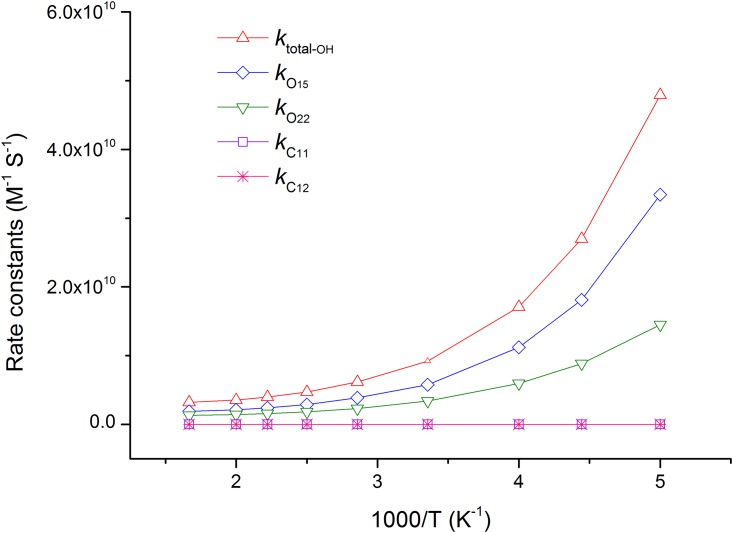
The calculated ICVT/SCT rate constants for the reactions of SA with ·OH and the total rate constants (*k*_total-OH_) versus 1,000/T, from 200 K to 600 K, in water phase (in M^-1^ S^-1^). The HAT channels O_15h_ and O_22h_ have negative temperature dependence, the RAF channels C_11h_ and C_12h_ have positive temperature dependence.

The temperature dependence of rate constants against the reciprocal of temperature for each channel and total reaction are sketched in Fig 9. It is clear that channel O_15h_ always maintains the largest rate constant from 200–600 K. The second channel O_22h_ is also very important, and its ratio branching is increasingly higher with the temperature rising. In addition, the rate constants of HAT channels have negative temperature dependence, while the RAF channels have positive temperature dependence.

According to the above points of view, thermodynamics and kinetics: among the two feasible mechanisms, HAT is the major mechanism for the antioxidant activity of SA toward ·OH in vivo. Channel O_15h_ is the major pathway of all reactions, followed by channel O_22h_, which is also not negligible. Although the RAF mechanism is thermodynamically feasible, its contribution to the total reaction is negligible.

The theoretical total rate constant at 298 K computed here (9.20×10^9^ M^-1^ S^-1^) agrees well with the pulse radiolysis experiment about the ·OH-scavenging capability of SA conducted in neutral solution (*k* = 9.60×10^9^ M^-1^ S^-1^) [70], which further supports the reliability of the calculations in our work.

In aqueous solutions, the ·OH-scavenging activity of SA was higher than other hydroxycinnamic acids, including ferulic acid (4.55×10^9^ M^-1^ S^-1^) [71], caffeic acid (3.24×10^9^ M^-1^ S^-1^) [72] and gallic acid (7.02×10^8^ M^-1^ S^-1^) [21]. Therefore, SA is an excellent ·OH scavenger among the hydroxycinnamic acid antioxidants.

### Radicals scavenging by SA^-^

In the environment of physiological pH (7.4), SA primarily exist in anionic form with a dissociated COOH group [73–74]. In order to provide a detailed investigation on the radical scavenging activity of SA toward ·NO_2_ and ·OH, also the anionic form of sinapic acid has been taken into account, considering HAT and RAF mechanisms in aqueous.

For each radical, one HAT channel (O_15_ for ·NO_2_; O_15h_ for ·OH) and two RAF channels (C_11_, C_12_ for ·NO_2_; C_11h_, C_12h_ for ·OH) were considered. Under the M05-2X/6-311++G(d,p) level, SA^-^ also has a planar stricture, the optimized geometries of SA^-^, TSs and PCs of all channels are gathered in S5 Fig. The Δ*H*, Δ*G* and Δ*E*+ZPE of all water-phase channels were also calculated and listed in Table 5.

**Table 5:** The reaction enthalpies (Δ*H*), reaction Gibbs energies (Δ*G*) and energy barrier heights with ZPE corrections (Δ*E*+ZPE), at 298 K, for the reactions of SA^-^ with ·NO_2_ and ·OH in water phase (in kJ/mol).

SA^-^+·NO_2_	Δ*H*_sol_	Δ*G*_sol_	Δ*E*_sol_+ZPE	SA^-^+·OH	Δ*H*_sol_	Δ*G*_sol_	Δ*E*_sol_+ZPE
**O**_**15**_	-14.73	-34.77	19.35	**O**_**15h**_	-176.21	-192.99	-10.12
**C**_**11**_	6.37	62.16	35.89	**C**_**11h**_	-161.61	-96.31	-8.73
**C**_**12**_	-36.34	10.43	15.98	**C**_**12h**_	-134.18	-113.51	-8.03

For scavenging ·NO_2_, according to the Table 5, the channel C_12_ has the lowest energy barrier height, but the Δ*G* of this channel is positive, meaning that it is could not occur spontaneously. While the channel O_15_, with a second lower energy barrier height, is the largest exergonic channel, hence it is the major channel of SA with ·NO_2_. The Δ*G* values of 62.16 and 10.43 kJ/mol for the RAF channels C_11_ and C_12_ indicate that this mechanism is quite unfavored also when the antioxidant is present in the anionic form, and thus it can be concluded that both SA and SA^-^ do not follow the RAF process when scavenging ·NO_2_ radicals.

For scavenging ·OH, all channels are exothermic and exoergic, indicating both HAT and RAF mechanisms are feasible. Among them, the channel O_15h_ has a absolute dominance, as it is the most exothermic and exoergic channel, and having the lowest energy barrier height. These results are in agreement with the scavenging activity of neutral SA toward ·OH.

Considering the largest contribution channel O_15_/O_15h_, the energy barrier heights of SA^-^ scavenging ·NO_2_ and ·OH are much higher than those of SA, thus the rate constants of SA^-^ scavenging both radicals should be lower than the rate constants of SA scavenging ·NO_2_ and ·OH.

## Conclusions

In this work, we carried out a systematic study on the radical scavenging activities of SA and SA^-^ toward ·NO_2_ and ·OH in aqueous simulated media using DFT and direct dynamic method.

For SA/SA^- ^scavenging,·NO_2_, only HAT reactions in water phase are thermodynamically feasible. The H-abstraction reaction from site O_15_ is the major channel of all reactions, while the RAF mechanism is not useful.

For SA/SA^-^ scavenging ·OH, both HAT and RAF mechanisms are very feasible thermodynamically and kinetically. In the HAT mechanism, the most active site is also the -O_15_H group in benzene ring. The RAF mechanism is weaker than HAT, and the activities of two channels (C_11h_ and C_12h_) are similar.

The reactions of SA eliminating ·NO_2_ and ·OH take place predominantly by the HAT mechanism (Γ>99%), especially via the channel O_15_/O_15h_. For the reactions of SA with ·OH, the site O_22h_ on the carboxyl group is also a significantly active site. Agreeing with our points, the DFT study from Galano et al. reported that the main mechanism of SA scavenging ·OOH should be HAT with the largest contribution (Γ≈99.9%), and the most important active site is the phenolic group of SA to scavenge ·OOH [22].

The total rate constants of SA scavenging ·NO_2_ and ·OH are 1.30×10^8^ and 9.20×10^9^ M^-1^ S^-1^ respectively, in water phase, at 298 K; and are 1.10×10^8^ and 8.20×10^9^ M^-1^ S^-1^ respectively, at 310 K. The rate constants in pulse radiolytic experiments are 7.20×10^8^ and 9.60×10^9^ M^-1^ S^-1^, respectively. The total rate constant of *k*_total-NO2_ is small than *k*_total-OH_, due to the high activity of ·OH, which is a reasonable result, but they are still very fast. Thus we state SA can efficiently scavenge ·NO_2_ and ·OH radicals in vivo.

## Supporting Information

S1 Fig The comparison of geometries between the TS optimized under SMD model and the TS optimized in the gas-phase. (TIF)

S2 Fig The transition state geometries for the reactions of SA with ·OH. (TIF)

S3 Fig The spin density plots for the products of SA scavenging ·NO_2_ and ·OH. (TIF)

S4 Fig The optimized geometries of reactants and products in aqueous solution for the reactions of SA with ·NO_2_ and ·OH. (TIF)

S5 Fig The optimized geometries of SA^-^, TSs and PCs for the reactions of SA^-^ with ·NO_2_ and ·OH. (TIF)

S1 Table The energy barrier heights obtained by optimization of TS under SMD model and the energy barrier heights obtained by the single point calculation with SMD model based on the gas-phase optimized geometries of TS. (DOC)

S2 Table The geometry coordinates of all species optimized at M05-2X/6-311++G(d,p) level. (DOC)

## References

[1] Andreasen MF, Landbo AK, Christensen LP, Hansen A, Meyer AS (2001) Antioxidant effects of phenolic rye (Secale cereale L.) extracts, monomeric hydroxycinnamates, and ferulic acid dehydrodimers on human low-density lipoproteins. Journal of Agricultural and Food Chemistry 49: 4090–4096. 11513715 10.1021/jf0101758

[2] Herrmann K, Nagel CW (1989) Occurrence and content of hydroxycinnamic and hydroxybenzoic acid compounds in foods. Critical Reviews in Food Science and Nutrition 28: 315–347. 2690858 10.1080/10408398909527504

[3] Thiyama U, Stockmanna H, Feldeb TZ, Schwarza K (2006) Antioxidative effect of the main sinapic acid derivatives from rapeseed and mustard oil by-products. European Journal of Lipid Science and Technology 108: 239–248.

[4] Yoon BH, Jung JW, Lee JJ, Cho YW, Jang CG, Jin C, et al (2007) Anxiolytic-like effects of sinapic acid in mice. Life Science 81: 234–240. 10.1016/j.lfs.2007.05.007 17570441

[5] Yun KJ, Koh DJ, Kim SH, Park SJ, Ryu JH, Kim DG, et al (2008) Anti-inflammatory effects of sinapic acid through the suppression of inducible nitric oxide synthase, cyclooxygase-2, and proinflammatory cytokines expressions via nuclear factor-kB inactivation. Journal of Agricultural and Food Chemistry 56: 10265–10272. 10.1021/jf802095g 18841975

[6] Niwa T, Doi U, Kato Y, Osawa T (1999) Inhibitory mechanism of sinapinic acid against peroxynitrite-mediated tyrosine nitration of protein in vitro. Federation of European Biochemical Societies 459: 43–46. 10.1016/s0014-5793(99)01216-8 10508914

[7] Zou Y, Kim AR, Kim JE, Choi JS, Chung HY (2002) Perox-ynitrite scavenging ability of sinapic acid (3,5-Dimethoxy-4-hydroxycinnamic Acid) isolated from brassica juncea. Journal of Agricultural and Food Chemistry 50: 5884–5890. 12358454 10.1021/jf020496z

[8] Rice-Evans C, Miller N, Paganga G (1997) Antioxidant properties of phenolic compounds. Trends in Plant Science 2: 152–159.

[9] Cai YZ, Luo Q, Sun M, Corke H (2004) Antioxidant activity and phenolic compounds of 112 traditional chinese medicinal plants associated with anticancer. Life Science 74: 2157–2184. 10.1016/j.lfs.2003.09.047 PMC7126989 14969719

[10] Silva FAM, Borges F, Guimaraes C, Lima JLFC, Matos C, Reis S (2000) Phenolic acids and derivatives: studies on the relationship among structure, radical scavenging activity, and physicochemical parameters. Journal of Agricultural and Food Chemistry 48: 2122–2126. 10888509 10.1021/jf9913110

[11] Cheng JC, Dai F, Zhou B, Yang L, Liu ZL (2007) Antioxidant activity of hydroxycinnamic acid derivatives in human low density lipoprotein: mechanism and structure-activity relationship. Food Chemistry 104: 132–139.

[12] Zheng W, Wang SY (2001) Antioxidant activity and phenolic compounds in selected herbs. Journal of Agricultural and Food Chemistry 49: 5165–5170. 11714298 10.1021/jf010697n

[13] Cuvelier ME, Richard H, Berset C (1992) Comparison of the antioxidative activity of some acid-phenols: structure-activity relationship. Bioscience, Biotechnology, and Biochemistry 56: 324–325.

[14] Valentao P, Fernandes E, Carvalho F, Andrade PB, Seabra RM, Bastos ML (2001) Antioxidant activity of centaurium erythraea infusion evidenced by its superoxide radical scavenging and xanthine oxidase inhibitory activity. Journal of Agricultural and Food Chemistry 49: 3476–3479. 11453794 10.1021/jf001145s

[15] Cuvelier ME, Richard H, Berset C (1992) Comparison of the antioxidative activity of some acid-phenols: structure-activity relationship. Bioscience, Biotechnology, and Biochemistry 56:324–325.

[16] Valko M, Rhodes CJ, Moncol J, Izakovic M, Mazur M (2006) Free radicals, metals and antioxidants in oxidative stress-induced cancer. Chemico-Biological Interactions 160: 1–40. 16430879 10.1016/j.cbi.2005.12.009

[17] Willcox JK, Ash SL, Catignani GL (2004) Antioxidants and prevention of chronic disease. Critical Reviews in Food Science and Nutrition 44: 275–295. 15462130 10.1080/10408690490468489

[18] Street DA, Comstock GW, Salkeld RM, Schuep W, Klag MJ (1994) Serum antioxidants and myocardial infarction. Are low levels of carotenoids and alpha-tocopherol risk factors for myocardial infarction? Circulation 90: 1154–1161. 8087925 10.1161/01.cir.90.3.1154

[19] Chisolm GM (1991) Antioxidants and atherosclerosis. A current assessment. Clinical Cardiology 14: 25–30. 2044256 10.1002/clc.4960141304

[20] Halliwell B (2001) Role of Free Radicals in the Neurodegenerative Diseases: Therapeutic Implications for Antioxidant Treatment. Drugs & Aging 18: 685–716. 11599635 10.2165/00002512-200118090-00004

[21] Marino T, Galano A, Russo N (2014) Radical scavenging ability of gallic acid toward OH and OOH radicals. reaction mechanism and rate constants from the density functional theory. The Journal of Physical Chemistry B 118: 10380–10389. 10.1021/jp505589b 25119432

[22] Galano A, Francisco-Marquez M, Alvarez-Idaboy JR (2011) Mechanism and kinetics studies on the antioxidant activity of sinapinic acid. Physical Chemistry Chemical Physics 23: 11199–11205. 10.1039/c1cp20722a 21566849

[23] Urbaniak A, Molski M, Szeląg M (2012) quantum-chemical calculations of the antioxidant properties of trans-p-coumaric acid and trans-sinapinic acid. Computational in Science and Technology 18: 1–12.

[24] Saqib M, Iqbal S, Mahmood A, Akram R (2016) Theoretical investigation for exploring the antioxidant potential of chlorogenic acid: a density functional theory study. International Journal of Food Properties 19: 745–751.

[25] Chen YZ, Xiao H, Zheng J, Liang G (2015) Structure-thermodynamics-antioxidant activity relationships of selected natural phenolic acids and derivatives: an experimental and theoretical evaluation. Plos One: 1–20. 10.1371/journal.pone.0121276 PMC4372407 25803685

[26] Filipovic M, Markovic Z, Dorovic J, Markovic JD, Lucic B, Amic D (2015) QSAR of the free radical scavenging potency of selected hydroxybenzoic acids and simple phenolics. Comptes Rendus Chimie 18: 492–498.

[27] Saqib M, Iqbal S, Naeem S, Mahmood A (2013) DFT for exploring the antioxidant potential of homogentisic and orsellinic acids. Pakistan Journal of Pharmaceutical Sciences 26: 1209–1214. 24191328

[28] Urbaniak A, Szeląg M, Molski M (2013) Theoretical investigation of stereochemistry and solvent influence on antioxidant activity of ferulic acid. Computational and Theoretical Chemistry 1012: 33–40.

[29] Nenadis N, Tsimidou MZ (2012) Contribution of DFT computed molecular descriptors in the study of radical scavenging activity trend of natural hydroxybenzaldehydes and corresponding acids. Food Research International 48: 538–543.

[30] Mohajeri A, Asemani SS (2009) Theoretical investigation on antioxidant activity of vitamins and phenolic acids for designing a novel antioxidant. Journal of Molecular Structure 930: 15–20.

[31] Leopoldini M, Chiodo SG, Russo N, Toscano M (2011) Detailed investigation of the OH radical quenching by natural antioxidant caffeic acid studied by quantum mechanical models. January of Chemical Theory and Computation 7: 4218–4233. 10.1021/ct200572p 26598362

[32] Garzon A, Bravo I, Barbero AJ, Albaladejo J (2014) Mechanistic and kinetic study on the reactions of coumaric acids with reactive oxygen species: a DFT approach. Journal of Agricultural and Food Chemistry 62:9705–9710. 10.1021/jf5011148 25166496

[33] Meo FD, Lemaur V, Cornil J, Lazzaroni R, Duroux JL, Olivier T, et al (2013) Free radical scavenging by natural polyphenols: atom versus electron transfer. The Journal of Physical Chemistry A 117: 2082–2092. 10.1021/jp3116319 23418927

[34] Pryor WA, Lightsey JW (1981) Mechanisms of nitrogen dioxide reaction: initiation of lipid peroxidation and the production of nitrous acid. Science 214: 435–437. 17730242 10.1126/science.214.4519.435

[35] Pryor WA, Lightsey JW, Church DF (1982) Reaction of nitrogen dioxide with alkenes and polyunsaturated fatty acids: addition and hydrogen-abstraction mechanisms. Journal of the American Chemistry Society 104: 6685–6692.

[36] Csallany AS, Ayaz KL (1978) Long-term NO_2_ exposure of mice in the presence and absence of vitamin E. I. Effect on body weights and lipofuscin in pigments. Archives of Environmental Health 33: 285–291. 736610 10.1080/00039896.1978.10667349

[37] Draganic IG, Draganic ZD (1971) The radiation chemistry of water. Academic Press: New York.

[38] Leopoldini M, Pitarch IP, Russo N, Toscano M (2004) Structure, conformation, and electronic properties of apigenin, luteolin, and taxifolin antioxidants. A first principle theoretical study. The January of Physical Chemistry A 108: 92–96.

[39] Wright JS, Johnson ER, Di Labio GA (2001) Predicting the activity of phenolic antioxidants: theoretical method, analysis of substituent effects, and application to major families of antioxidants. January of the American Chemical Society 123: 1173–1183. 10.1021/ja002455u 11456671

[40] Urbaniak A, Molski M, Szeląg M (2012) Quantum-chemical calculations of the antioxidant properties of trans-p-coumaric acid and trans-sinapinic acid. Computational Methods in Science Technology 18: 117–128.

[41] Frisch MJ, Trucks GW, Schlegel HB, Scuseria GE, Robb MA, Cheeseman JR, et al Gaussian 09, Revision A. 02: Gaussian, Inc., Wallingford CT, 2009.

[42] Zhao Y, Schultz NE, Truhlar DG (2006) Design of density functionals by combining the method of constraint satisfaction with parametrization for thermochemistry, thermochemical kinetics, and noncovalent interactions. Journal Chemistry Theory Computation 2: 364–382. 10.1021/ct0502763 26626525

[43] Aruoma OI, Murcia A, Butler J, Halliwell B (1993) Evaluation of the antioxidant and prooxidant actions of gallic acid and its derivatives. Journal of Agricultural and Food Chemistry 41: 1880–1885.

[44] Velez E, Quijano J, Notario R, Pabon E, Murillo J, Leal J, et al (2009) Computational study of stereospecifity in the thermal elimination reaction of menthyl benzoate in the gas phase. Journal Physical Organic Chemistry 22: 971–977.

[45] Galano A, Alvarez-Idaboy JR (2009) Guanosine + OH radical reaction in aqueous solution: a reinterpretation of the UV-vis data based on thermodynamic and kinetic calculations. Organic Letters 11: 5114–5117. 10.1021/ol901862h 19839587

[46] Black G, Simmie JM (2010) Barrier heights for H-atom abstraction by HO_2_ from n-butanol-a simple yet exacting test for model chemistries? Journal Computation Chemistry 31: 1236–1248. 10.1002/jcc.21410 19882733

[47] Furuncuoglu T, Ugur I, Degirmenci I, Aviyente V (2010) Role of chain transfer agentls in free radical polymerization kinetics. Macromolecules 43: 1823–1835.

[48] Zhao Y, Schultz NE, Truhlar DG (2006) Design of density functionals by combining the method of constraint satisfaction with parameterization for thermochemistry, thermochemical kinetics, and noncovalent interactions. Journal of Chemical Theory Computation 2: 364–382. 10.1021/ct0502763 26626525

[49] Zavala-Oseguera C, Alvarez-Idaboy JR, Merino G, Galano A (2009) OH radical gas phase reactions with aliphatic ethers: a variational transition State Theory Study. The Journal of Physical Chemistry A 113: 13913–13920. 10.1021/jp906144d 19908880

[50] Perez-Gonzalez A, Galano A (2011) OH radical scavenging activity of edaravone: mechanism and kinetics. The Journal of Physical Chemistry B 115: 1306–1314. 10.1021/jp110400t 21190324

[51] Vega-Rodriguez A, Alvarez-Idaboy JR (2009) Quantum chemistry and TST study of the mechanisms and branching ratios for the reactions of OH with unsaturated aldehydes. Physical Chemistry Chemical Physics 11: 7649–7658. 19950504 10.1039/b906692f

[52] Zhao Y, Truhlar DG (2008) How well can new-generation density functionals describe the energetics of bond-dissociation reactions producing radicals? The Journal Physical Chemistry A 112: 1095–1099. 10.1021/jp7109127 18211046

[53] Marenich AV, Cramer CJ, Truhlar DG (2009) Universal solvation model based on solute electron density and on a continuum model of the solvent defined by the bulk dielectric constant and atomic surface tensions. The Journal Physical Chemistry A 113: 6378–6396. 10.1021/jp810292n 19366259

[54] Agnihotri N, Mishra PC (2011) Scavenging mechanism of curcumin toward the hydroxyl radical: a theoretical study of reactions producing ferulic acid and vanillin. The Journal of Physical Chemistry A 115:14221–14232. 10.1021/jp209318f 22035040

[55] Vallet V, Wahlgren U, Schimmelpfennig B, Szabo Z, Grenthe I (2001) The mechanism for water exchange in [UO2(H2O)5]2+ and [UO2(oxalate)2(H2O)]2-, as studied by quantum chemical methods. Journal of the American Chemical Society 123: 11999–12008. 11724608 10.1021/ja015935+

[56] Gao JY, Yang X, Kim CK, Xue Y (2012) Theoretical studies on the chemical decomposition of 5-aza-2'-deoxycytidine: DFT study and Monte Carlo simulation. Theoretical Chemistry Accounts 131: 1108–1123.

[57] Okuno Y (1997) Theoretical investigation of the mechanism of the baeyer-villiger reaction in nonpolar solvents. Chemistry-A European Journal 3: 212–218. 10.1002/chem.19970030208 24022950

[58] Ardura D, Lopez R, Sordo TL (2005) Relative gibbs energies in solution through continuum models: effect of the loss of translational degrees of freedom in bimolecular reactions on gibbs energy barriers. Journal of Physical Chemistry B 109: 23618–23623. 10.1021/jp0540499 16375339

[59] Galano A, Alvarez-Idaboy JR (2013) A computational methodology for accurate predictions of rate constants in solution: application to the assessment of primary antioxidant activity. Journal of Computational Chemistry 34: 2430–2445. 10.1002/jcc.23409 23939817

[60] Garrett BC, Truhlar DG (1980) Improved canonical variational theory for chemical reaction rates. Classical mechanical theory and applications to collinear reactions. The Journal Physical Chemistry 84: 805–812.

[61] Lu D, Truong TN, Melissas VS, Lynch GC, Liu YP, Garrett BC, et al (1992) POLYRATE 4: a new version of a computer program for the calculation of chemical reaction rates for polyatomics. Computer Physics Communications 71: 235–262.

[62] Corchado JC, Chuang Y-Y, Fast PL, Hu W-P, Liu Y-P, Lynch GC, et al (2007) Polyrate, version 9.7. Department of Chemistry and Super-computer Institute, University of Minnesota, Minneapolis

[63] Chuang YY, Cramer CJ, Truhlar DG (1998) The interface of electronic structure and dynamics for reactions in solution. International Journal of Quantum Chemistry 70:887–896.

[64] Bakalbassis EG, Chatzopoulou A, Melissas VS, Tsimidou M, Tsolaki M, Vafiadis A (2001) Ab initio and density functional theory studies for the explanation of the antioxidant activity of certain phenolic acids. Lipids 36: 181–191. 11269699 10.1007/s11745-001-0705-9

[65] van Acker SABE, de Groot MJ, van den Berg DJ, Tromp MNJL, den Kelder GDO, van der Vijgh WJF, et al (1996) A quantum chemical explanation of the antioxidant activity of flavonoids. Chemical Research in Toxicology 9: 1305–1312. 8951233 10.1021/tx9600964

[66] Chen W, Guo P, Song J, Cao W, Bian J (2006) The ortho hydroxy-amino group: another choice for synthesizing novel antioxidants. Bioorganic & Medicinal Chemistry Letters 16: 3582–3585. 16621555 10.1016/j.bmcl.2006.03.091

[67] Zhang ZE, Yao S, Lin WZ, Wang WF, Jin YZ, Lin NY (1998) Mechanism of reaction of nitrogen dioxide radical with hydroxycinnamic Acid derivatives: a pulse radiolysis study. Free radical Research 29: 13–16. 9733017 10.1080/10715769800300021

[68] Prutz WA, Monig H, Butler J, Land EJ (1985) Reactions of nitrogen dioxide in aqueous model systems: oxidation of tyosine units in peptides and proteins. Archives of Biochemistry and Biophysics 243: 125–134. 4062299 10.1016/0003-9861(85)90780-5

[69] Forni LG, Victor O, Mora-Arellano VO, Packer JE, Willson RI (1986) Nitrogen dioxide and related free radicals: electron transfer reactions with organic compounds in solutions containing nitrite or nitrate. Journal of the Chemical Society, Perkin Transactions 2 1: 1–6.

[70] Wang WF, Luo J, Yao SD, Lian ZR, Zhang JS, Lin NY, et al (1993) Interaction of phenolic antioxidants and hydroxyl radicals. Radiation Physics and Chemistry 42: 985–987.

[71] Scott B, Butler J, Halliwell B, Aruoma OI (1993) Evaluation of the antioxidant actions of ferulic acid and catechins. Free Radical Research Communications 19:241–253. 7507456 10.3109/10715769309056512

[72] Kono Y, Kobayashi K, Tagawa S, Adachi K, Ueda A, Sawa Y, et al (1997) Antioxidant activity of polyphenolics in diets rate constants of reactions of chlorogenic acid and caffeic acid with reactive species of oxygen and nitrogen. Biochimica et Biophysica Acta 1335: 335–342. 9202196 10.1016/s0304-4165(96)00151-1

[73] Smyk B (2003) Fluorescence study of sinapic acid interaction with bovine serum albumin and egg albumin. Journal of Fluorescence 13: 349–356.

[74] Smyk B, Drabent R (1989) Spectroscopic investigation of the equilibria of the ionic forms of sinapic acid. ANALYST 114: 723–726.

