# Supplementary figures and images for: Kinetic Reaction Mechanism of Sinapic Acid Scavenging NO2 and OH Radicals: A Theoretical Study
Source: PLoS One. 2016 Sep 13;11(9):e0162729. doi: 10.1371/journal.pone.0162729 (PMC5021273; doi:10.1371/journal.pone.0162729)

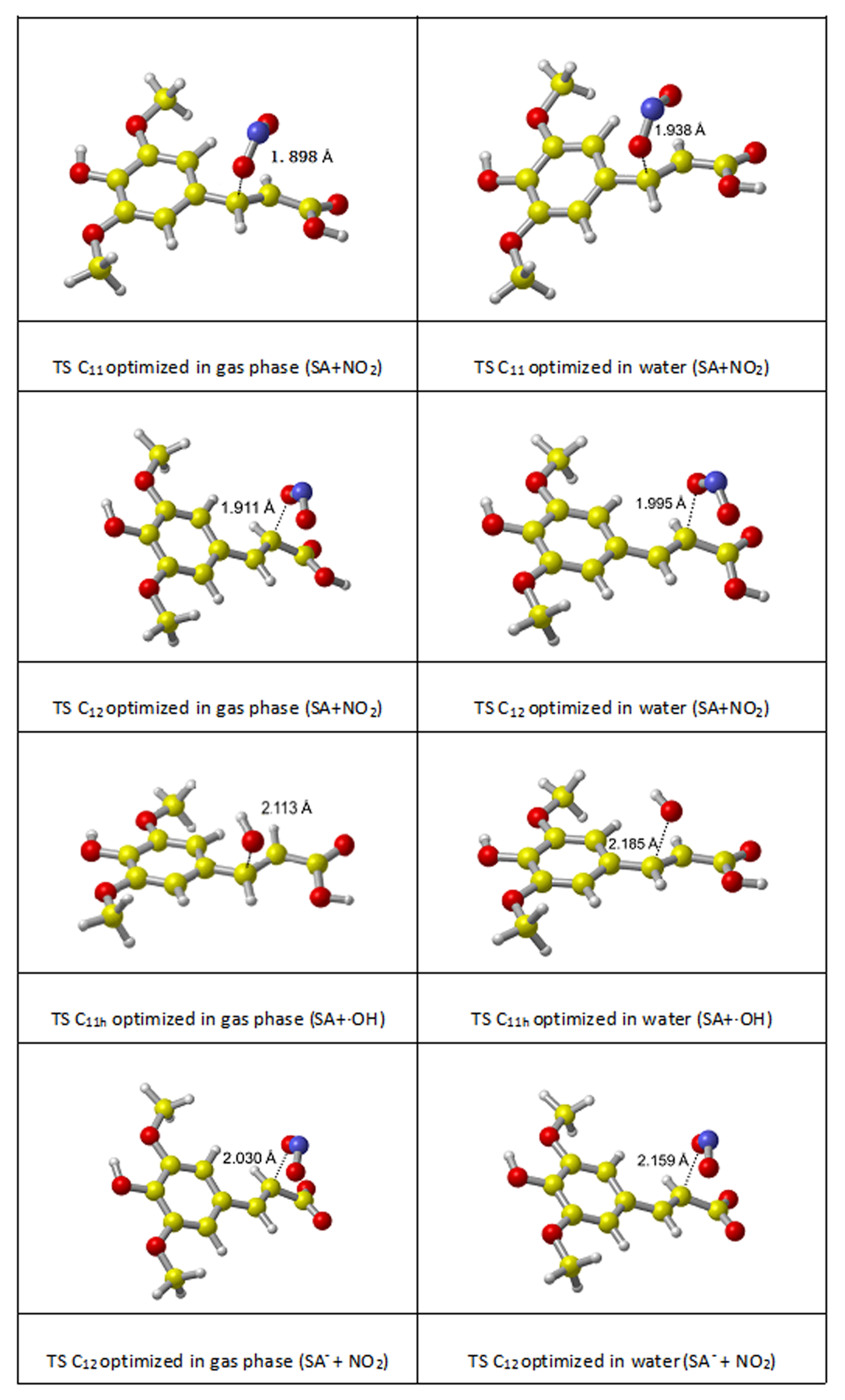

Supplement: S1 Fig — (TIF) [file pone.0162729.s001.tif]

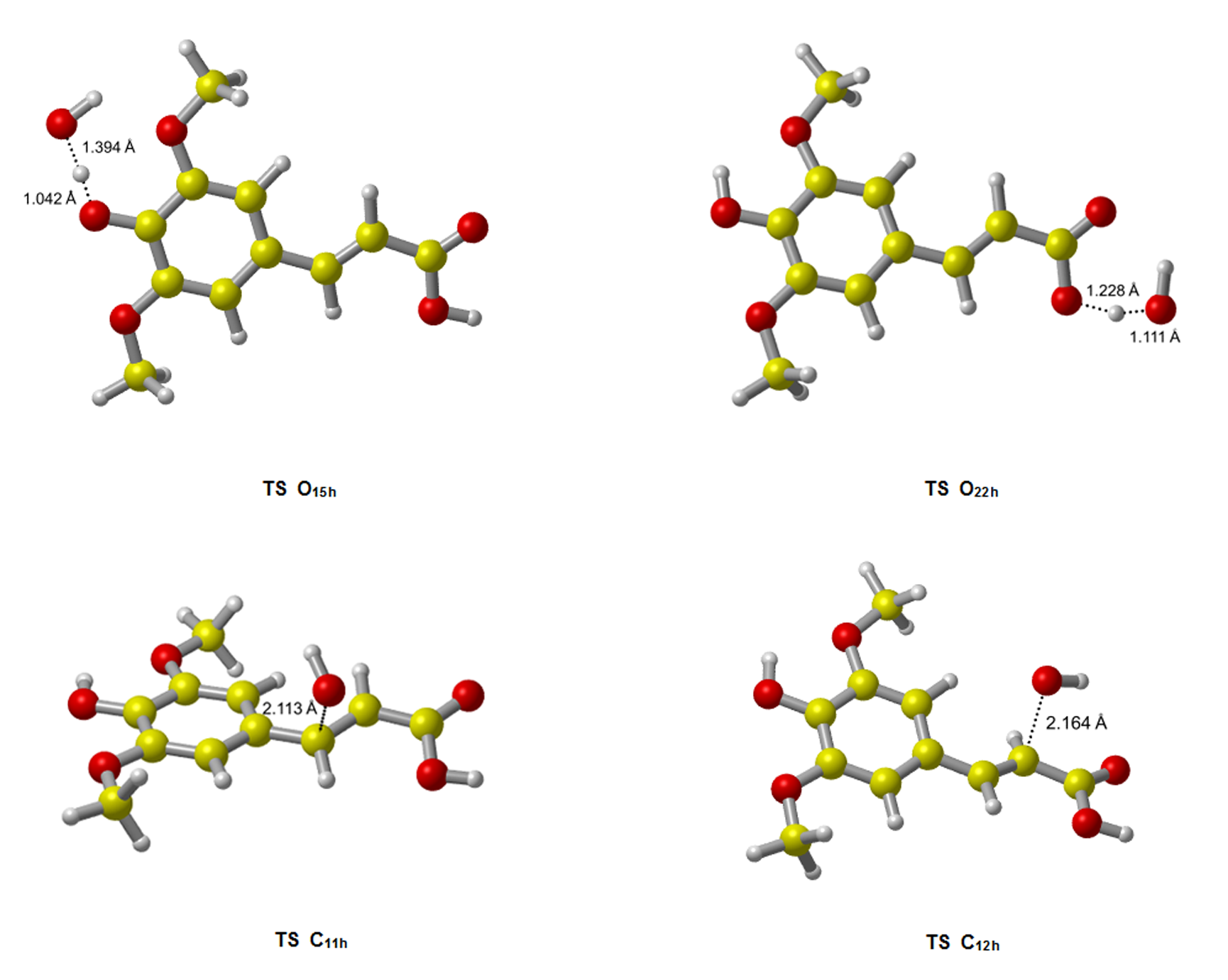

Supplement: S2 Fig — (TIF) [file pone.0162729.s002.tif]

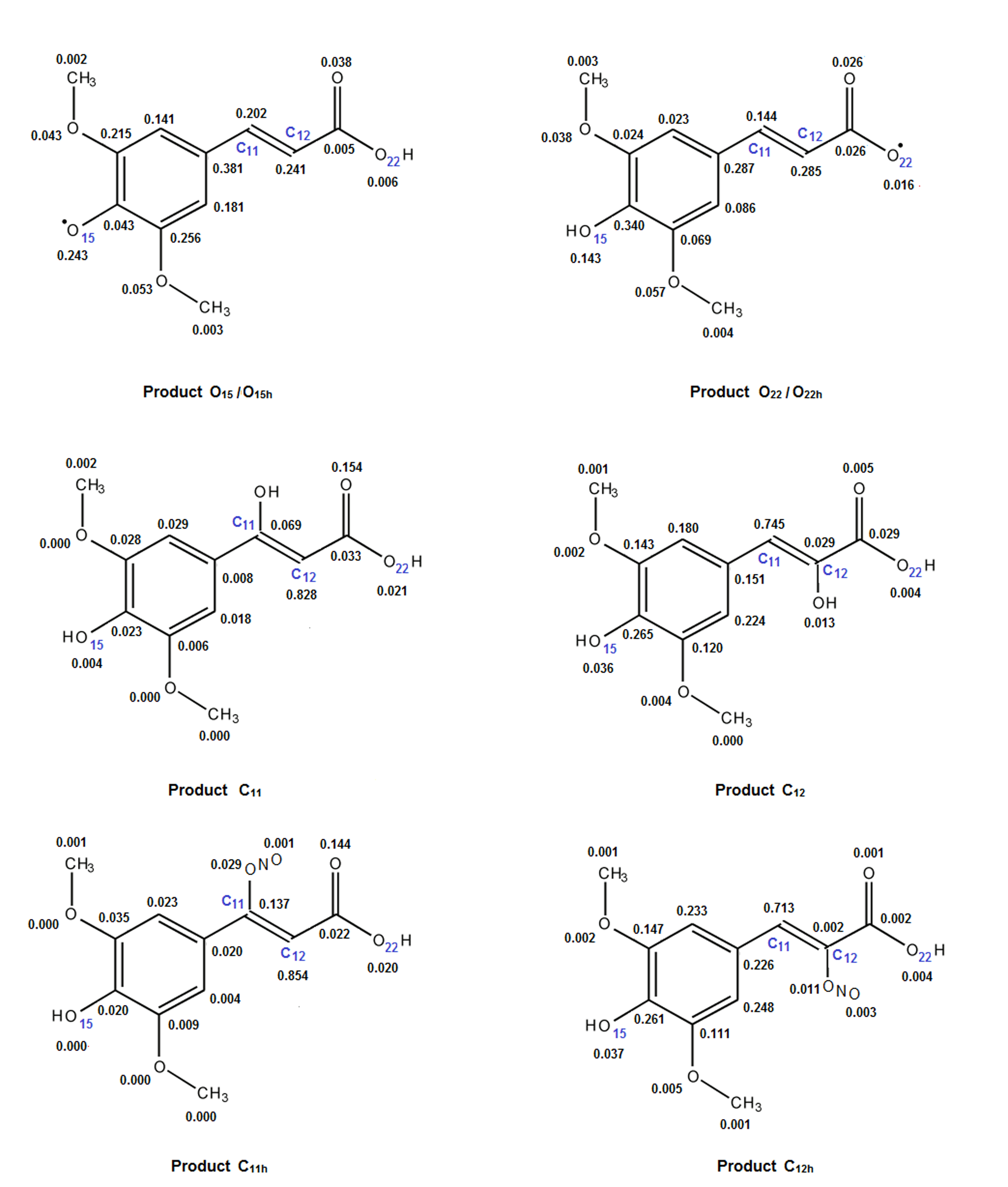

Supplement: S3 Fig — (TIF) [file pone.0162729.s003.tif]

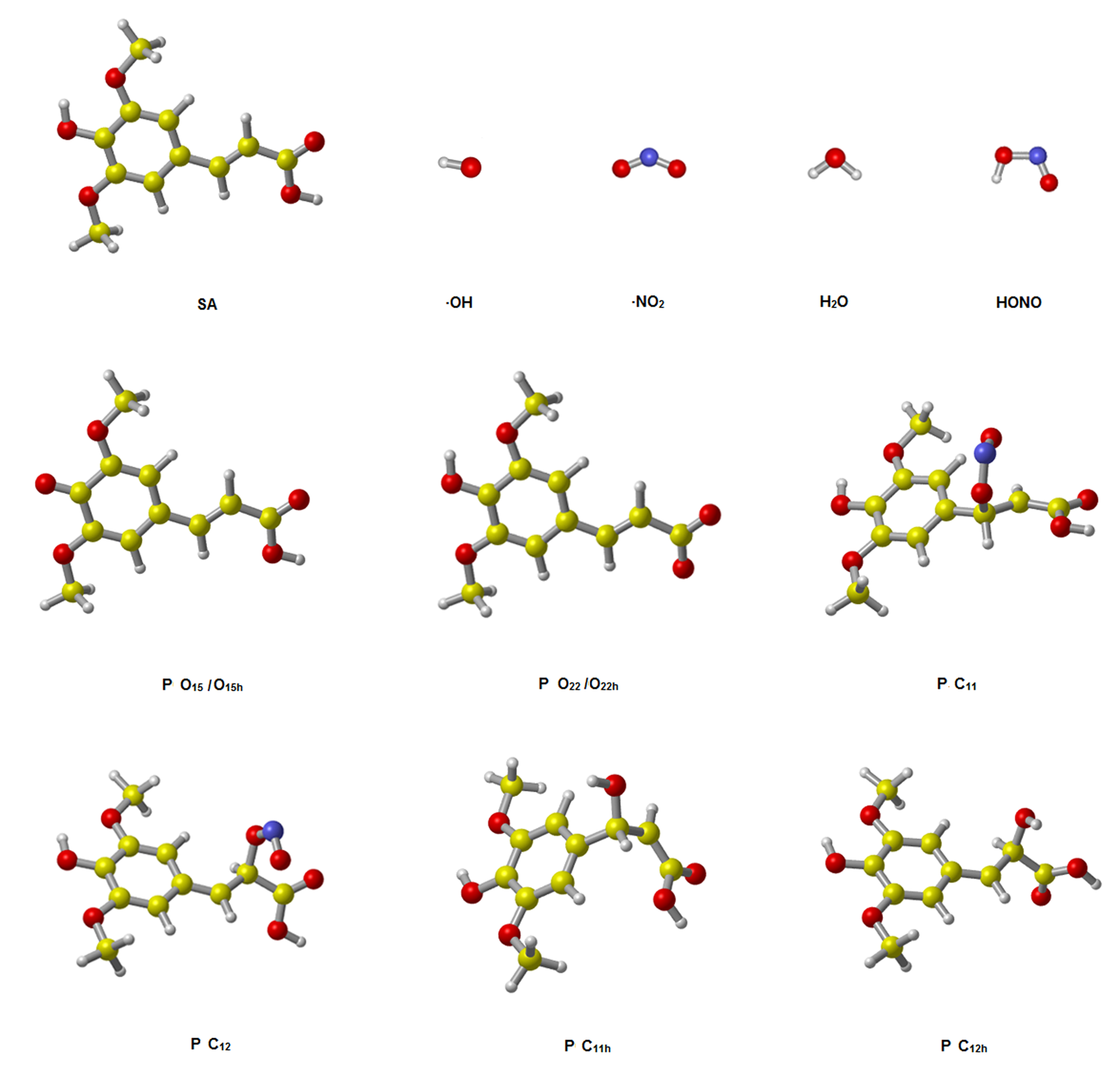

Supplement: S4 Fig — (TIF) [file pone.0162729.s004.tif]

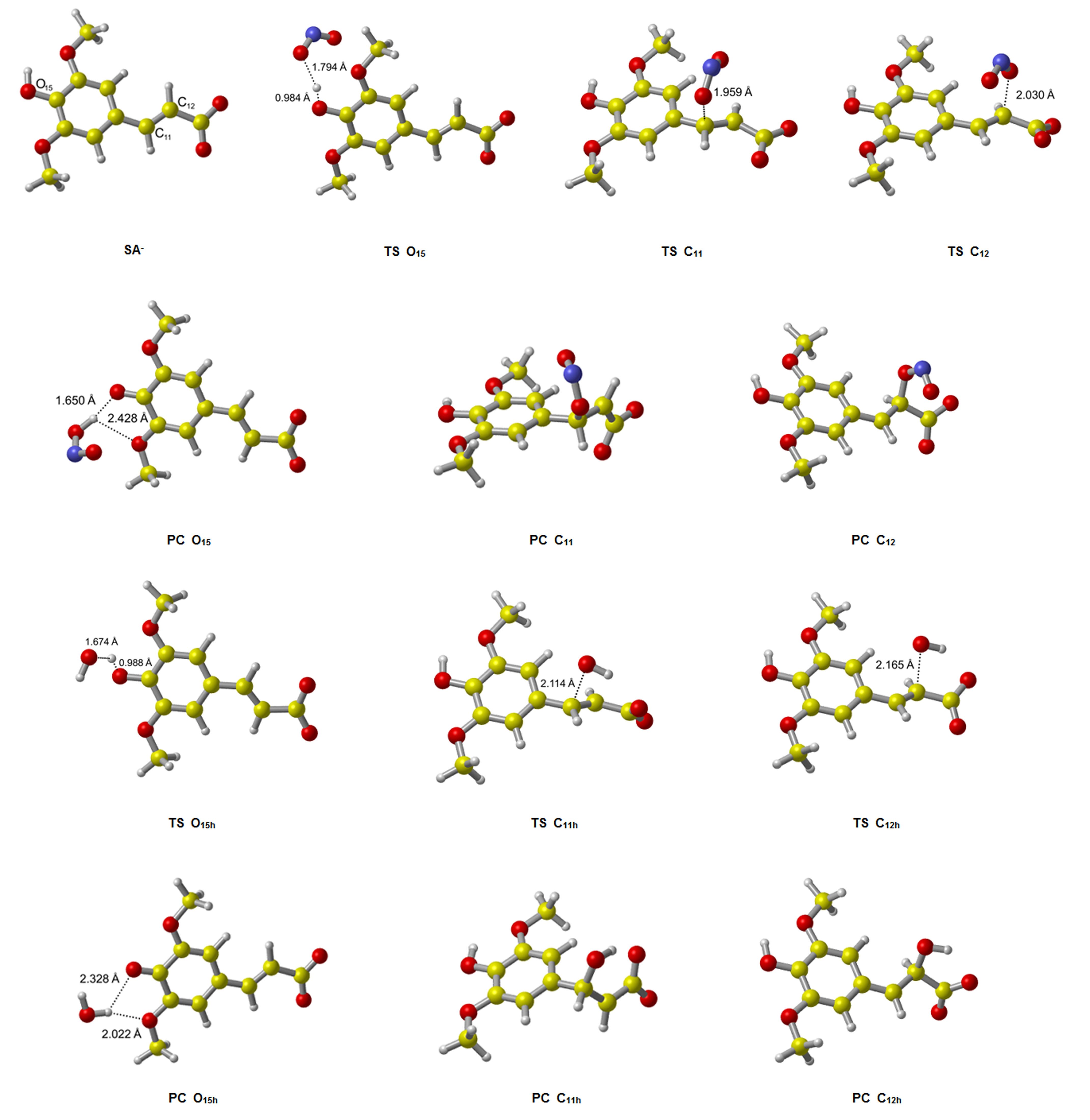

Supplement: S5 Fig — (TIF) [file pone.0162729.s005.tif]
