# Supplementary material for: Kinetic Reaction Mechanism of Sinapic Acid Scavenging NO2 and OH Radicals: A Theoretical Study
Source: PLoS One. 2016 Sep 13;11(9):e0162729. doi: 10.1371/journal.pone.0162729 (PMC5021273; doi:10.1371/journal.pone.0162729)
Supplement: S1 Table — (DOC) [file pone.0162729.s006.doc]

Table S1. The energy barrier heights obtained by optimization of TS under SMD model and the energy barrier heights obtained by the single point calculation with SMD model based on the gas-phase optimized geometries of TS
site	ΔEsol a	ΔEsol b	
C11 (SA+NO2)	44.84 kJ/mol	42.20 kJ/mol	
C12 (SA+NO2)	32.15 kJ/mol	31.07 kJ/mol	
C11h (SA+·OH)	-3.20 kJ/mol	-5.02 kJ/mol	
C12 (SA- + NO2)	15.98 kJ/mol	10.46 kJ/mol	
C11h (SA- + ·OH)	-8.73 kJ/mol	-12.58 kJ/mol	

a The energy of TS obtained by single point energy calculation with SMD model.
b The energy of TS obtained by optimization in aqueous solution.
