# Supplementary material for: Kinetic Reaction Mechanism of Sinapic Acid Scavenging NO2 and OH Radicals: A Theoretical Study
Source: PLoS One. 2016 Sep 13;11(9):e0162729. doi: 10.1371/journal.pone.0162729 (PMC5021273; doi:10.1371/journal.pone.0162729)
Supplement: S2 Table — (DOC) [file pone.0162729.s007.doc]

Table S2. The geometry coordinates of all species optimized at M05-2X/6-311++G(d,p) level

Reactants:
SA in gas phase
C	-4.2231950	-0.0190210	-0.0628750
C	-0.3840920	-0.2445400	 0.0829300
C	 0.1152890	 1.0604510	 0.0773750
C	 0.4989500	-1.3263890	 0.0475230
C	 1.4837260	 1.2589770	 0.0234800
H	-0.5595490	 1.8995380	 0.1309780
C	 1.8741870	-1.1226620	-0.0172700
H	 0.0953340	-2.3273870	 0.0563750
C	 2.3718740	 0.1805100	-0.0249100
H	-2.0912470	-1.5585280	 0.2991840
C	-1.8171980	-0.5272700	 0.1091130
C	-2.8045870	 0.3515600	-0.0981750
H	-2.6340940	 1.3925660	-0.3303350
O	 2.8025920	-2.1050660	-0.0755810
O	 3.7038220	 0.3887920	-0.0736580
H	 3.8488200	 1.3417090	-0.0608960
C	 2.3380020	-3.4440600	-0.0502660
H	 1.7991400	-3.6508470	 0.8767680
H	 3.2265230	-4.0647700	-0.1020320
H	 1.6962900	-3.6514510	-0.9092090
O	-5.1293430	 0.7451000	-0.2824030
O	-4.4483580	-1.3194100	 0.2367960
H	-5.4039110	-1.4439190	 0.2077570
C	 1.2874000   3.6325070	 0.0786570
H	 1.9637760	 4.4803270	 0.0645300
H	 0.6232550	 3.6777640	-0.7857130
H	 0.7051840	 3.6375560	 1.0014560
O	 2.1073290	 2.4739660	 0.0145510

SA in water
 C   4.2154830  -0.0347540   0.0129440
 C   0.3810440  -0.2454900  -0.0108690
 C  -0.1111140   1.0648490  -0.0101530
 C  -0.5002300  -1.3308560  -0.0072290
 C  -1.4797560   1.2696970  -0.0066580
 H   0.5623150   1.9078370  -0.0158520
 C  -1.8738100  -1.1159220   0.0003460
 H  -0.0999610  -2.3337900  -0.0094710
 C  -2.3649350   0.1871730  -0.0004760
 H   2.0617520  -1.5947410  -0.0569240
 C   1.8106930  -0.5414590  -0.0173380
 C   2.8075830   0.3539360   0.0241170
 H   2.6423380   1.4205290   0.0751180
 O  -2.8167430  -2.0992990   0.0071590
 O  -3.7129250   0.3874850   0.0043550
 H  -3.8811210   1.3398350   0.0052610
 C  -2.3460870  -3.4508230   0.0341010
 H  -1.7610560  -3.6694420  -0.8586780
 H  -3.2364610  -4.0705910   0.0525360
 H  -1.7512990  -3.6290380   0.9292700
 O   5.1323970   0.7654690   0.0887650
 O   4.4434780  -1.3546640  -0.0908440
 H   5.4006490  -1.5023480  -0.0897820
 C  -1.2593960   3.6478700  -0.0153270
 H  -1.9357330   4.4960360  -0.0164970
 H  -0.6367590   3.6699260   0.8782340
 H  -0.6412130   3.6642030  -0.9121170
 O  -2.0962440   2.4855410  -0.0096660

SA- in gas phase
 C	-4.3368990	-0.1946690	-0.0347980
 C	-0.4217150	-0.2867410	 0.0655450
 C 	 0.0541470	 1.0282390	 0.0643320
 C   0.5021490	-1.3346830	 0.0348890
 C	 1.4178010	 1.2632630	 0.0151690
 H	-0.6505970	 1.8426790	 0.1135770
 C	 1.8729180	-1.0907130	-0.0134280
 H	 0.1227200	-2.3452140	 0.0411730
 C	 2.3402890	 0.2191360	-0.0254790
 H	-2.1304990	-1.6362940	 0.2998480
 C	-1.8566770	-0.6043770	 0.1018110
 C	-2.8664970	 0.2392270	-0.1080470
 H	-2.7075780	 1.2870630	-0.3419420
 O	 2.8311120	-2.0609520	-0.0488320
 O	 3.6805820	 0.4769320	-0.0657290
 H	 3.7711230  1.4351330	-0.0588280
 C	 2.3840900	-3.4014980	-0.0382850
 H	 1.8224620	-3.6222310	 0.8718930
 H	 3.2807060	-4.0138880	-0.0709750
 H	 1.7611360	-3.6158910	-0.9093060
 O	-5.1463430	 0.7432360	-0.2003400
 O	-4.5488340	-1.4042170	 0.1801790
 C	 1.1379360	 3.6283560	 0.0690530
 H	 1.7773800	 4.5062240	 0.0602530
 H	 0.4731640	 3.6459670	-0.7959010
 H	 0.5451190	 3.6114460	 0.9847990
 O	 2.0046840	 2.5089720	 0.0123270

SA- in water
 C   4.2972790  -0.1870180   0.0058590
 C   0.4151950  -0.2747300  -0.0127450
 C  -0.0530270   1.0436640  -0.0112510
 C  -0.4913860  -1.3379560  -0.0085300
 C  -1.4187790   1.2777880  -0.0056520
 H   0.6378490   1.8723100  -0.0179480
 C  -1.8615820  -1.0938460  -0.0003450
 H  -0.1149090  -2.3500120  -0.0111400
 C  -2.3275300   0.2169890   0.0000770
 H   2.0743610  -1.6608970  -0.0655590
 C   1.8458910  -0.6016080  -0.0214770
 C   2.8660740   0.2595700   0.0216070
 H   2.7109600   1.3297560   0.0743620
 O  -2.8238830  -2.0608570   0.0054950
 O  -3.6752090   0.4467360   0.0055200
 H  -3.8194260   1.4025960   0.0046150
 C  -2.3782870  -3.4203330   0.0332410
 H  -1.7973200  -3.6512750  -0.8591970
 H  -3.2798890  -4.0239800   0.0516120
 H  -1.7871380  -3.6102830   0.9285860
 O   5.1650620   0.7281900   0.0895730
 O   4.5584050  -1.4164210  -0.0901360
 C  -1.1450970   3.6518380  -0.0151820
 H  -1.8013620   4.5158610  -0.0156990
 H  -0.5196620   3.6613640   0.8767950
 H  -0.5283710   3.6538370  -0.9132550
 O  -2.0078260   2.5091750  -0.0063010

NO2 in gas phase
 N   0.0000000   0.0000000   0.3131080
 O   0.0000000   1.0901310  -0.1369850
 O   0.0000000  -1.0901310  -0.1369850

NO2 in water
 N   0.0000000   0.0000000   0.3147300
 O   0.0000000   1.0890320  -0.1376940
 O   0.0000000  -1.0890320  -0.1376940

·OH in gas phase
 O   0.0000000   0.0000000   0.1077750
 H   0.0000000   0.0000000  -0.8621990

·OH in water
 O   0.0000000   0.0000000   0.1080820
 H   0.0000000   0.0000000  -0.8646590

Transition States of SA scavenging ·NO2
TS O15
 C	 4.9673800	-0.5605550	 0.2261180
 C	 1.1592450	-0.1651440	-0.1625290
 C	 0.8737080	 1.2106850	-0.0727190
 C	 0.1371570	-1.0878290	-0.3984840
 C	-0.4210610	 1.6625720	-0.2076990
 H	 1.6705690	 1.9073820	 0.1283940
 C	-1.1621000	-0.6435230	-0.5770810
 H	 0.3740170	-2.1390770	-0.4585650
 C	-1.4766940	 0.7370920	-0.4670890
 H	 2.6096220	-1.7613910	-0.0013970
 C	 2.5144410	-0.6823950	-0.0203400
 C	 3.6345690	 0.0444270	 0.0761240
 H	 3.6507480	 1.1237590	 0.0493410
 O	-2.2267720	-1.4130630	-0.8151480
 O	-2.6925740	 1.1712240	-0.5807060
 H	-3.4644690	 0.4872520	-0.3416200
 C	-2.0937210	-2.8237670	-0.6524210
 H	-1.4228100	-3.2296630	-1.4098290
 H	-3.0909420	-3.2277450	-0.7859520
 H	-1.7313290	-3.0474290	 0.3502890
 O	 5.9844080	 0.0748650	 0.3243780
 O	 4.9571740	-1.9106230	 0.2453710
 H	 5.8733010	-2.1945990	 0.3475500
 C	 0.1788360	 3.9148620	 0.1799380
 H	-0.3485330	 4.8605600	 0.2324760
 H	 0.6556760	 3.7023300	 1.1378930
 H	 0.9270600	 3.9523850	-0.6134000
 O	-0.8121540	 2.9389380	-0.1123660
 N	-4.5570570	-0.9511690	 1.0603070
 O	-3.5151550	-1.1221010	 1.6277690
 O	-4.6541850	-0.1660060	 0.1106740

TS C11
 C	 3.7281400	-0.3877490	-0.9524150
 C	-0.0347990	-0.3620040	-0.1192120
 C	-0.4460550	 0.9694330	-0.1952840
 C	-0.9735240	-1.3906990	-0.0532810
 C	-1.8023930	 1.2478920	-0.2152130
 H	 0.2836710	 1.7636340	-0.2021060
 C	-2.3352400	-1.1052640	-0.0873050
 H	-0.6296630	-2.4111330	 0.0202900
 C	-2.7525380	 0.2241470	-0.1694400
 H	 1.6106240	-1.7702550	 0.0173860
 C	 1.3908110	-0.7165690	-0.0920910
 C	 2.3439870	 0.0427280	-0.7889170
 H	 2.0947870	 0.9773460	-1.2609610
 O	-3.3224440	-2.0269070	-0.0425090
 O	-4.0697980	 0.5091100	-0.1929440
 H	-4.1599370	 1.4678730	-0.2364450
 C	-2.9417250	-3.3880640	 0.0701980
 H	-2.3497140	-3.6995410	-0.7925360
 H	-3.8682340	-3.9514950	 0.0984270
 H	-2.3796940	-3.5599320	 0.9900240
 O	 4.5542090	 0.2120730	-1.5930700
 O	 4.0071410	-1.5459450	-0.3118180
 H	 4.9361970	-1.7435630	-0.4803260
 C	-1.4638230	 3.6050840	-0.2887250
 H	-2.0915640	 4.4893700	-0.3118660
 H	-0.8459180	 3.6126880	 0.6098160
 H	-0.8328650	 3.5768270	-1.1778890
 O	-2.3521540	 2.4944290	-0.2731870
 N	 2.2427950	 0.5851860	 2.1133600
 O	 2.4098830	 1.4682010	 1.3244580
 O	 1.8051260	-0.5211770	 1.7494160

TS C12
 C	 3.6737570	-0.3704100	-0.9225410
 C	-0.1105130	-0.3824220	-0.2669000
 C	-0.5118610	 0.9657450	-0.2725680
 C	-1.0771070	-1.3979750	-0.1539400
 C	-1.8557770	 1.2660990	-0.1864530
 H	 0.2268150	 1.7510270	-0.3034560
 C	-2.4249510	-1.0893000	-0.0780940
 H	-0.7456780	-2.4249210	-0.1336730
 C	-2.8213430	 0.2542430	-0.0976060
 H 	 1.5283060	-1.7831290	-0.1373510
 C	 1.2673420	-0.7558320	-0.3462920
 C	 2.3012710	 0.1326530	-0.6278280
 H 	 2.0941030	 1.0492600	-1.1589980
 O	-3.4279500	-1.9875890	 0.0214790
 O	-4.1260190	 0.5620050	-0.0148980
 H	-4.2018630	 1.5236130	-0.0244220
 C	-3.0713520	-3.3597940	 0.0687590
 H	-2.5569980	-3.6588730	-0.8463470
 H	-4.0056580	-3.9035720	 0.1570240
 H	-2.4416100	-3.5673410	 0.9357450
 O	 4.4702320	 0.2393750	-1.5807770
 O	 3.9295150	-1.5749500	-0.3878780
 H	 4.8353870	-1.8054870	-0.6282910
 C	-1.4892340	 3.6195720	-0.2091680
 H	-2.1049590	 4.5116370	-0.1712120
 H	-0.8173860	 3.5951290	 0.6493270
 H	-0.9156650	 3.6057170	-1.1365250
 O	-2.3904730	 2.5193260	-0.1661740
 N	 2.5397340	 0.6419350	 2.0375510
 O	 2.3262750	-0.5277160	 2.1035090
 O	 2.7241310	 1.1842900	 0.9111280

Transition States of SA scavenging ·OH
TS O15h
 C	 4.5320090	-0.3822920	 0.0668960
 C	 0.7456750	 0.3134420	-0.0049150
 C	 0.0883350	-0.9180560	-0.0642030
 C	 0.0046560	 1.5001000	-0.0120740
 C	-1.2921410	-0.9527860	-0.1518600
 H	 0.6561470	-1.8335980	-0.0362430
 C	-1.3799790	 1.4696560	-0.0855600
 H	 0.5301770	 2.4412880	 0.0407580
 C	-2.0563070	 0.2329750	-0.1694010
 H	 2.5909360	 1.4209800	 0.2031610
 C 	 2.1995910	 0.4197370	 0.0676240
 C	 3.0802980	-0.5835790	-0.0240930
 H	 2.7963460	-1.6144050	-0.1754390
 O	-2.1730770	 2.5567250	-0.0994180
  O	-3.3685460	 0.2392660	-0.2815390
 H	-3.8890390	-0.6093680	 0.0246100
 C	-1.5501250	 3.8288170	-0.0260370
 H	-0.9899790	 3.9341230	 0.9048490
 H	-2.3577010	 4.5523770	-0.0502550
 H	-0.8888910	 3.9870100	-0.8798620
 O	 5.3423340	-1.2697690	-0.0148430
 O	 4.9004050	 0.9054620	 0.2533790
 H	 5.8640110	 0.9126570	 0.2921980
 C	-1.3258140	-3.3304420	-0.2798930
 H	-2.0850940	-4.1001890	-0.3744110
 H	-0.6831320	-3.3406600	-1.1604170
 H	-0.7350780	-3.5009750	 0.6207460
 O	-2.0290350	-2.0950890	-0.1963470
 O	-4.5649030	-1.5317020	 0.8216710
 H	-3.9433150	-2.2738120	 0.8281800

TS C11h
 C	 4.0622050	 0.2241120	-0.2032810
 C	 0.2226520	-0.1705840	-0.1967330
 C	-0.2942640	 1.1240160	-0.1212220
 C	-0.6356790	-1.2711720	-0.1906450
 C	-1.6669540	 1.2943730	-0.0313020
 H	 0.3631300	 1.9777510	-0.1666080
 C	-2.0107420	-1.0952090	-0.0919370
 H	-0.2062750	-2.2604190	-0.2309670
 C	-2.5317000	 0.1989040	-0.0119140
 H	 1.9667120	-1.3568890	-0.7324260
 C	 1.6635490	-0.4238390	-0.2801760
 C	 2.6336900	 0.4972570	-0.0155350
 H	 2.4203320	 1.4622560	 0.4196860
 O	-2.9212170	-2.0931050	-0.0670640
 O	-3.8660580	 0.3767850	 0.0717430
 H	-4.0311640	 1.3256960	 0.1071670
 C	-2.4339340	-3.4231380	-0.1451000
 H	-1.8986440	-3.5865210	-1.0821640
 H	-3.3114330	-4.0600600	-0.1105200
 H	-1.7814350	-3.6491470	 0.7000880
 O	 4.9371810	 0.9890000	 0.1152540
 O	 4.3175520	-0.9659280	-0.7813150
 H	 5.2768380	-1.0571320	-0.8182950
 C	-1.5215870	 3.6742950	-0.0117490
 H	-2.2172820	 4.5048150	 0.0422420
 H	-0.8384730	 3.7105790	 0.8380080
 H	-0.9610140	 3.7223910	-0.9461480
 O	-2.3132430	 2.4958730	 0.0344260
 O	 2.1663930	-1.2871920	 1.5812440
 H	 1.7304210	-0.6308710	 2.1431830

TS C12h
 C	 3.9678430	-0.4696150	-0.3266700
 C	 0.1448890	-0.4210640	-0.0209540
 C	-0.2129160	 0.9322750	-0.0742370
 C	-0.8446770	-1.4121470	 0.0357330
 C	-1.5528430	 1.2667960	-0.0953600
 H	 0.5579550	 1.6852750	-0.0219690
 C	-2.1883540	-1.0688490	-0.0056000
 H	-0.5429570	-2.4468980	 0.0954460
 C	-2.5462620	 0.2817000	-0.0744810
 H	 1.7623100	-1.8058210	 0.3706080
 C	 1.5319310	-0.8154860	-0.0012840
 C	 2.5673700	-0.0179000	-0.3841870
 H	 2.4136510	 0.8840700	-0.9543840
 O	-3.2189500	-1.9438010	 0.0255920
 O	-3.8474510	 0.6242580	-0.0933370
 H	-3.8938450	 1.5873640	-0.1148120
 C	-2.8996040	-3.3212540	 0.1257420
 H	-2.3168260	-3.6494330	-0.7371680
 H	-3.8499150	-3.8440030	 0.1439970
 H	-2.3498540	-3.5272930	 1.0460930
 O	 4.9081660	 0.2260880	-0.6225150
 O	 4.1228800	-1.7352800	 0.1064260
 H	 5.0720240	-1.9054520	 0.1349510
 C	-1.1129120	 3.6049150	-0.0878770
 H	-1.6993450	 4.5174480	-0.0849650
 H	-0.5016240	 3.5510510	 0.8132960
 H	-0.4767090	 3.5762420	-0.9732050
 O	-2.0518120	 2.5360870	-0.1171610
 O	 2.7233320	 1.3425680	 1.2912400
 H	 3.6090290	 1.6218630	 1.0151990

Products of SA scavenging ·NO2 in gas phase
HONO
 N   0.0000000   0.5402390  0.0000000
 O   1.0715370   0.0649710  0.0000000
 O  -0.9993800  -0.3803500  0.0000000
 H  -0.5772560  -1.2586430  0.0000000

P O15
 C   4.1701570  -0.0828940  0.0000070
 C   0.3334320  -0.2474530  0.0000040
 C  -0.1387770   1.0870260  0.0000480
 C  -0.5709060  -1.3309820 -0.0000310
 C  -1.4829650   1.3439450  0.0000210
 H   0.5705650   1.8982020  0.0001030
 C  -1.9263950  -1.1113170 -0.0000380
 H  -0.1694320  -2.3331050 -0.0000520
 C  -2.4768760   0.2534130 -0.0000610
 H   1.9984750  -1.6128420 -0.0000550
 C   1.7446410  -0.5597230  -0.0000170
 C   2.7594920   0.3236990  0.0000010
 H   2.6192400   1.3938820  0.0000890
 O  -2.8649560  -2.0569720  -0.0000460
 O  -3.6778560   0.4697170  -0.0000220
 C  -2.4426590  -3.4134850  0.0001330
 H  -1.8576870  -3.6328470 -0.8942810
 H  -3.3519280  -4.0040960  0.0002420
 H  -1.8576320  -3.6325820  0.8945750
 O   5.0931180   0.6906830  0.0002110
 O   4.3565350  -1.4216560 -0.0002130
 H   5.3098450  -1.5681620 -0.0001850
 C  -1.1783110   3.6834640 -0.0000730
 H  -1.8290260   4.5507420 -0.0001620
 H  -0.5533870   3.6883950  0.8944090
 H  -0.5534090   3.6881820 -0.8945710
 O  -2.0406690   2.5544850  0.0000610

P O22
 C  -4.2339540  -0.2028220  0.0000010
 C  -0.4227510  -0.2514980 -0.0000020
 C   0.0680860   1.0579060 -0.0001330
 C   0.4673720  -1.3287810  0.0001190
 C   1.4355810   1.2637740 -0.0001400
 H  -0.6109940   1.8952620 -0.0002360
 C   1.8424800  -1.1176620  0.0001200
 H   0.0693200  -2.3319900  0.0002530
 C   2.3312830   0.1891630 -0.0000220
 H  -2.1168600  -1.6007420  0.0000780
 C  -1.8482660  -0.5490560  0.0000110
 C  -2.8645120   0.3231110 -0.0000360
 H  -2.7414640   1.3969770 -0.0000880
 O   2.7785530  -2.0922190  0.0003230
 O   3.6608310   0.4050010 -0.0000280
 H   3.8017250   1.3586650 -0.0000320
 C   2.3245370  -3.4358060 -0.0002350
 H   1.7351490  -3.6482870  0.8936130
 H   3.2196920  -4.0485670 -0.0004050
 H   1.7352770  -3.6475620 -0.8943370
 O  -5.1813050   0.7256770 -0.0001250
 O  -4.5938480  -1.3578790  0.0001430
 C   1.2354200   3.6394610  0.0002630
 H   1.9136320   4.4859870  0.0004030
 H   0.6115780   3.6666050 -0.8941510
 H   0.6119210   3.6660030  0.8949330
 O   2.0556900   2.4795330 -0.0002760

P C11
 C   3.7403390  -0.2924110 -0.9864840
 C  -0.0048940  -0.3215400  0.0022870
 C  -0.4513850   0.9938100 -0.1039260
 C  -0.9170280  -1.3738520  0.0070470
 C  -1.8148750   1.2331450 -0.2015560
 H   0.2489200   1.8123230 -0.0937270
 C  -2.2843130  -1.1280950 -0.0941940
 H  -0.5524930  -2.3863350  0.0926340
 C  -2.7370010   0.1870270 -0.2000970
 H   1.5979570  -1.7251340 -0.0674940
 C   1.4551290  -0.6644220  0.1306440
 C   2.3727160   0.1097730 -0.7401120
 H   2.0394280   0.9890540 -1.2640940
 O  -3.2436600  -2.0805360 -0.1029710
 O  -4.0608240   0.4336150 -0.2988130
 H  -4.1742790   1.3891450 -0.3519720
 C  -2.8286850  -3.4301130  0.0223780
 H  -2.1889320  -3.7206470 -0.8132100
 H  -3.7385890  -4.0206250  0.0058950
 H  -2.3045390  -3.5905770  0.9663110
 O   4.5136670   0.3193260 -1.6864480
 O   4.0827270  -1.4405590 -0.3564930
 H   5.0037500  -1.6211370 -0.5788640
 C  -1.5387990   3.6012370 -0.2490870
 H  -2.1899650   4.4671500 -0.3028850
 H  -0.9805780   3.6147190  0.6876610
 H  -0.8507230   3.6053210 -1.0953690
 O  -2.3921490   2.4657470 -0.3046500
 N   2.1667510   0.5146190  2.1540600
 O   2.0968800   1.4668170  1.4631430
 O   1.8753040  -0.6522770  1.5631450

P C12
 C   3.6691700  -0.4065120 -0.7613100
 C  -0.1352950  -0.4028760 -0.1645600
 C  -0.5419170   0.9528260 -0.2424960
 C  -1.1276340  -1.4068490 -0.0191510
 C  -1.8845630   1.2597110 -0.1917760
 H   0.1969710   1.7355640 -0.3021400
 C  -2.4685430  -1.0827040  0.0290550
 H  -0.8071810  -2.4352240  0.0444920
 C  -2.8602180   0.2621350 -0.0618280
 H   1.4802700  -1.8219930 -0.1183250
 C   1.2150270  -0.7830780 -0.2270610
 C   2.3089030   0.2012170 -0.4482080
 H   2.0720090   0.8471790 -1.2981200
 O  -3.4819340  -1.9687340  0.1596550
 O  -4.1677340   0.5821210 -0.0113660
 H  -4.2335320   1.5421020 -0.0731660
 C  -3.1370320  -3.3388940  0.2695250
 H  -2.6171580  -3.6821960 -0.6269660
 H  -4.0760340  -3.8719980  0.3741090
 H  -2.5147870  -3.5138100  1.1493010
 O   4.7191010   0.0579320 -0.4308830
 O   3.5489390  -1.5014530 -1.5278930
 H   4.4426550  -1.8061150 -1.7314470
 C  -1.5040880   3.6078380 -0.3323160
 H  -2.1138530   4.5050210 -0.3431200
 H  -0.8410260   3.6201460  0.5333680
 H  -0.9192080   3.5479440 -1.2510220
 O  -2.4119710   2.5176330 -0.2484270
 N   2.7949050   0.6964360  1.8549300
 O   2.9834460  -0.4568600  1.8901410
 O   2.4556130   1.1795410  0.6136830

Products of SA scavenging ·NO2 in water
HONO
 N   0.0000000  0.5340830   0.0000000
 O   1.0764440  0.0580350   0.0000000
 O  -0.9995430 -0.3669430   0.0000000
 H  -0.6152070 -1.2673160   0.0000000

P O15
 C   4.1701570 -0.0828940   0.0000070
 C   0.3334320 -0.2474530   0.0000040
 C  -0.1387770  1.0870260   0.0000480
 C  -0.5709060 -1.3309820  -0.0000310
 C  -1.4829650  1.3439450   0.0000210
 H   0.5705650  1.8982020   0.0001030
 C  -1.9263950 -1.1113170  -0.0000380
 H  -0.1694320 -2.3331050  -0.0000520
 C  -2.4768760  0.2534130  -0.0000610
 H   1.9984750 -1.6128420  -0.0000550
 C   1.7446410 -0.5597230  -0.0000170
 C   2.7594920  0.3236990   0.0000010
 H   2.6192400  1.3938820   0.0000890
 O  -2.8649560 -2.0569720  -0.0000460
 O  -3.6778560  0.4697170  -0.0000220
 C  -2.4426590 -3.4134850   0.0001330
 H  -1.8576870 -3.6328470  -0.8942810
 H  -3.3519280 -4.0040960   0.0002420
 H  -1.8576320 -3.6325820   0.8945750
 O   5.0931180  0.6906830   0.0002110
 O   4.3565350 -1.4216560  -0.0002130
 H   5.3098450 -1.5681620  -0.0001850
 C  -1.1783110  3.6834640  -0.0000730
 H  -1.8290260  4.5507420  -0.0001620
 H  -0.5533870  3.6883950   0.8944090
 H  -0.5534090  3.6881820  -0.8945710
 O  -2.0406690  2.5544850   0.0000610

P O22
 C  -4.2748370 -0.2148040  -0.0009440
 C  -0.4122140 -0.2954110   0.0025240
 C   0.0277210  1.0547080   0.0070820
 C   0.5079110 -1.3675950  -0.0033920
 C   1.3654700  1.3200080   0.0058950
 H  -0.6831090  1.8645450   0.0138830
 C   1.8575260 -1.1185470  -0.0078650
 H   0.1310660 -2.3787050  -0.0053430
 C   2.3082950  0.2405850  -0.0032310
 H  -2.0446210 -1.6971620   0.0197320
 C  -1.8142720 -0.6392030   0.0074330
 C  -2.8353660  0.2373530  -0.0024240
 H  -2.6824590  1.3075890  -0.0218010
 O   2.8332860 -2.0192300  -0.0158770
 O   3.5913200  0.4737480  -0.0068860
 H   3.7654910  1.4325430  -0.0021820
 C   2.4526610 -3.4083240  -0.0176960
 H   1.8838980 -3.6340040   0.8823200
 H   3.3847310 -3.9606110  -0.0263310
 H   1.8706780 -3.6286570  -0.9106450
 O  -5.1292680  0.7013260  -0.1163430
 O  -4.5148030 -1.4410840   0.1147370
 C   1.1057780  3.6812670   0.0234320
 H   1.7802550  4.5292170   0.0284480
 H   0.4875590  3.6906900  -0.8723000
 H   0.4919060  3.6771880   0.9222530
 O   1.9547850  2.5173820   0.0127550

P C11
 C   3.7724740 -0.1706810  -0.9362620
 C  -0.0141140 -0.3252690  -0.0802370
 C  -0.4379760  1.0016650  -0.1156390
 C  -0.9381900 -1.3681870  -0.0874080
 C  -1.7998970  1.2711360  -0.1581430
 H   0.2701160  1.8141600  -0.1090050
 C  -2.3006240 -1.0889480  -0.1299110
 H  -0.5869300 -2.3893050  -0.0673590
 C  -2.7319860  0.2348530  -0.1643000
 H   1.5617980 -1.7314570  -0.3581920
 C   1.4414970 -0.7057900  -0.0119680
 C   2.3824240  0.1696710  -0.7502350
 H   2.0457800  1.0733730  -1.2307670
 O  -3.2861870 -2.0291250  -0.1523480
 O  -4.0717440  0.4955020  -0.2128800
 H  -4.1941760  1.4541040  -0.2386220
 C  -2.8779710 -3.3998410  -0.0999880
 H  -2.2656900 -3.6493530  -0.9661510
 H  -3.7952610 -3.9791170  -0.1182860
 H  -2.3314240 -3.6003240   0.8210010
 O   4.5604220  0.5346620  -1.5480840
 O   4.1343630 -1.3372520  -0.3782700
 H   5.0731500 -1.4919690  -0.5609080
 C  -1.4584470  3.6369650  -0.1799620
 H  -2.0902450  4.5185770  -0.2059380
 H  -0.8649100  3.6327990   0.7336380
 H  -0.8106200  3.6184170  -1.0556460
 O  -2.3524950  2.5178320  -0.1972860
 N   2.1098650  0.1749300   2.1758040
 O   2.0379340  1.2141460   1.6247240
 O   1.8522360 -0.8992490   1.4153830

P C12
 C   3.6410790 -0.3006880  -0.8328700
 C  -0.1402810 -0.3597430  -0.1656460
 C  -0.5701740  0.9898610  -0.2137110
 C  -1.1077070 -1.3904130  -0.0443690
 C  -1.9188930  1.2750650  -0.1570580
 H   0.1476510  1.7915370  -0.2876430
 C  -2.4530020 -1.0856210   0.0100370
 H  -0.7686960 -2.4145520  -0.0026400
 C  -2.8685300  0.2505280  -0.0481200
 H   1.4824330 -1.7673820  -0.1638080
 C   1.2175560 -0.7239450  -0.2344030
 C   2.3095640  0.2733510  -0.3801970
 H   2.0539380  1.0352650  -1.1195930
 O  -3.4578940 -1.9994270   0.1196800
 O  -4.1988000  0.5328480   0.0128440
 H  -4.3099850  1.4926630  -0.0292440
 C  -3.0745220 -3.3757800   0.2050200
 H  -2.5473860 -3.6811640  -0.6985480
 H  -4.0007820 -3.9327920   0.2987160
 H  -2.4494530 -3.5426570   1.0816630
 O   4.7144000  0.0792990  -0.4367720
 O   3.4749700 -1.2236550  -1.7733050
 H   4.3424410 -1.5258360  -2.0868660
 C  -1.5604550  3.6336140  -0.2781670
 H  -2.1840490  4.5213940  -0.2838350
 H  -0.8994530  3.6485200   0.5876750
 H  -0.9810270  3.5806240  -1.1992020
 O  -2.4634070  2.5248290  -0.1949980
 N   2.8844100  0.4580520   1.9554490
 O   3.0387990 -0.7007880   1.8446670
 O   2.5263950  1.0957230   0.8093940

Products of SA scavenging ·OH in water
H2O  
 O   0.0000000  0.0000000   0.1176680
 H   0.0000000  0.7601620  -0.4706720
 H   0.0000000 -0.7601620  -0.4706720

P O15h
 C   4.1701570 -0.0828940   0.0000070
 C   0.3334320 -0.2474530   0.0000040
 C  -0.1387770  1.0870260   0.0000480
 C  -0.5709060 -1.3309820  -0.0000310
 C  -1.4829650  1.3439450   0.0000210
 H   0.5705650  1.8982020   0.0001030
 C  -1.9263950 -1.1113170  -0.0000380
 H  -0.1694320 -2.3331050  -0.0000520
 C  -2.4768760  0.2534130  -0.0000610
 H   1.9984750 -1.6128420  -0.0000550
 C   1.7446410 -0.5597230  -0.0000170
 C   2.7594920  0.3236990   0.0000010
 H   2.6192400  1.3938820   0.0000890
 O  -2.8649560 -2.0569720  -0.0000460
 O  -3.6778560  0.4697170  -0.0000220
 C  -2.4426590 -3.4134850   0.0001330
 H  -1.8576870 -3.6328470  -0.8942810
 H  -3.3519280 -4.0040960   0.0002420
 H  -1.8576320 -3.6325820   0.8945750
 O   5.0931180  0.6906830   0.0002110
 O   4.3565350 -1.4216560  -0.0002130
 H   5.3098450 -1.5681620  -0.0001850
 C  -1.1783110  3.6834640  -0.0000730
 H  -1.8290260  4.5507420  -0.0001620
 H  -0.5533870  3.6883950   0.8944090
 H  -0.5534090  3.6881820  -0.8945710
 O  -2.0406690  2.5544850   0.0000610

P O22h
 C  -4.2748370 -0.2148040  -0.0009440
 C  -0.4122140 -0.2954110   0.0025240
 C   0.0277210  1.0547080   0.0070820
 C   0.5079110 -1.3675950  -0.0033920
 C   1.3654700  1.3200080   0.0058950
 H  -0.6831090  1.8645450   0.0138830
 C   1.8575260 -1.1185470  -0.0078650
 H   0.1310660 -2.3787050  -0.0053430
 C   2.3082950  0.2405850  -0.0032310
 H  -2.0446210 -1.6971620   0.0197320
 C  -1.8142720 -0.6392030   0.0074330
 C  -2.8353660  0.2373530  -0.0024240
 H  -2.6824590  1.3075890  -0.0218010
 O   2.8332860 -2.0192300  -0.0158770
 O   3.5913200  0.4737480  -0.0068860
 H   3.7654910  1.4325430  -0.0021820
 C   2.4526610 -3.4083240  -0.0176960
 H   1.8838980 -3.6340040   0.8823200
 H   3.3847310 -3.9606110  -0.0263310
 H   1.8706780 -3.6286570  -0.9106450
 O  -5.1292680  0.7013260  -0.1163430
 O  -4.5148030 -1.4410840   0.1147370
 C   1.1057780  3.6812670   0.0234320
 H   1.7802550  4.5292170   0.0284480
 H   0.4875590  3.6906900  -0.8723000
 H   0.4919060  3.6771880   0.9222530
 O   1.9547850  2.5173820   0.0127550

P C11h
 C  -3.2581130  0.0137810  -0.8988790
 C  -0.3948550 -0.2122390   0.6929590
 C   0.4165400 -1.2794720   0.3095600
 C   0.0519130  1.1016050   0.6036450
 C   1.6981760 -1.0132880  -0.1533540
 H   0.0490860 -2.2921560   0.3835510
 C   1.3344000  1.3587580   0.1249820
 H  -0.5964490  1.9082220   0.9113470
 C   2.1579960  0.3004070  -0.2512870
 H  -2.2182710  0.4229890   1.6071790
 C  -1.8118140 -0.4903210   1.1716120
 C  -2.6155840 -0.9083160  -0.0053400
 H  -2.6689870 -1.9570640  -0.2593950
 O   1.8803750  2.6007580  -0.0030030
 O   3.4189840  0.5726120  -0.7068790
 H   3.8510150 -0.2651990  -0.9198050
 C   1.0633360  3.7131210   0.3761690
 H   0.1601100  3.7501650  -0.2324010
 H   1.6671940  4.5958460   0.1933520
 H   0.8049670  3.6537660   1.4330520
 O  -3.8859420 -0.3272050  -1.8940960
 O  -3.1240650  1.3084140  -0.5529040
 H  -3.5896270  1.8535760  -1.2042730
 C   2.2177650 -3.3274840  -0.4341650
 H   3.0769070 -3.9030380  -0.7622690
 H   1.9814790 -3.5689890   0.6014910
 H   1.3637470 -3.5341550  -1.0779960
 O   2.6057470 -1.9533200  -0.5472020
 O  -1.8647060 -1.5595160   2.1120430
 H  -1.2828770 -1.3372230   2.8470830

P C12h
 C  -3.2581130  0.0137810  -0.8988790
 C  -0.3948550 -0.2122390   0.6929590
 C   0.4165400 -1.2794720   0.3095600
 C   0.0519130  1.1016050   0.6036450
 C   1.6981760 -1.0132880  -0.1533540
 H   0.0490860 -2.2921560   0.3835510
 C   1.3344000  1.3587580   0.1249820
 H  -0.5964490  1.9082220   0.9113470
 C   2.1579960  0.3004070  -0.2512870
 H  -2.2182710  0.4229890   1.6071790
 C  -1.8118140 -0.4903210   1.1716120
 C  -2.6155840 -0.9083160  -0.0053400
 H  -2.6689870 -1.9570640  -0.2593950
 O   1.8803750  2.6007580  -0.0030030
 O   3.4189840  0.5726120  -0.7068790
 H   3.8510150 -0.2651990  -0.9198050
 C   1.0633360  3.7131210   0.3761690
 H   0.1601100  3.7501650  -0.2324010
 H   1.6671940  4.5958460   0.1933520
 H   0.8049670  3.6537660   1.4330520
 O  -3.8859420 -0.3272050  -1.8940960
 O  -3.1240650  1.3084140  -0.5529040
 H  -3.5896270  1.8535760  -1.2042730
 C   2.2177650 -3.3274840  -0.4341650
 H   3.0769070 -3.9030380  -0.7622690
 H   1.9814790 -3.5689890   0.6014910
 H   1.3637470 -3.5341550  -1.0779960
 O   2.6057470 -1.9533200  -0.5472020
 O  -1.8647060 -1.5595160   2.1120430
 H  -1.2828770 -1.3372230   2.8470830

Transiton States of SA- scavenging ·NO2
TS O15
 C	 4.9676730	-0.9145170	 0.1971190
 C	 1.3008140	 0.3965490	-0.0890840
 C	 0.4434270	-0.6822290	-0.3580760
 C	 0.7581100	 1.6918380	 0.0131490
 C	-0.9056060	-0.4573410	-0.5366570
 H	 0.8498420	-1.6771630	-0.4386390
 C	-0.5974370	 1.9158110	-0.1363690
 H	 1.4345370	 2.5066940	 0.2203010
 C	-1.4564750	 0.8362410	-0.4195300
 H	 3.3142280	 1.1420500	 0.2703960
 C	 2.7358840	 0.2441130	 0.0770700
 C	 3.4422190	-0.8944560	 0.0170730
 H	 2.9838370	-1.8593180	-0.1701460
 O	-1.2027230	 3.1222740	-0.0317530
 O	-2.7600330	 1.0505040	-0.5547340
 H	-3.2771920	 0.2174880	-0.4757260
 C	-0.3782280	 4.2296380	 0.2824700
 H	 0.1176740	 4.0848560	 1.2441660
 H	-1.0422860	 5.0865520	 0.3366000
 H	 0.3706140	 4.3918010	-0.4951070
 O	 5.4678280	-2.0492600	 0.0667950
 O	 5.5015100	 0.1841500	 0.4401130
 C	-1.4388010	-2.7644460	-0.6824550
 H	-2.3395590	-3.3487860	-0.8401970
 H	-0.6939480	-3.0171590	-1.4383950
 H	-1.0453710	-2.9514330	 0.3165620
 O	-1.8330140	-1.4049910	-0.8072190
 N	-4.4161660	-1.7264490	 0.8548310
 O	-4.6150300	-0.8757270	 0.0092590
 O	-3.4321250	-1.7891550	 1.5398000

TS C11
 C	 3.8510280	-0.5798300	-0.9699150
 C	 0.0512640	-0.3488880	-0.0974690
 C	-0.3920540	 0.9647670	-0.2488960
 C	-0.8748710	-1.3871530	-0.0057060
 C	-1.7521450	 1.2128400	-0.3079600
 H	 0.3306320	 1.7631640	-0.2759700
 C	-2.2415600	-1.1307460	-0.0771780
 H	-0.5070660	-2.3941210	 0.1180340
 C	-2.6861930	 0.1797300	-0.2300230
 H	 1.7377550	-1.7263860	 0.1084840
 C	 1.4877240	-0.6834780	-0.0432310
 C	 2.4668830	 0.0208470	-0.7400050
 H	 2.2725450	 1.0003640	-1.1524390
 O	-3.2136510	-2.0815700	-0.0036910
 O	-4.0184210	 0.4498840	-0.2890680
 H	-4.0994720	 1.4059480	-0.3718430
 C	-2.7886200	-3.4160860	 0.1943110
 H	-2.1673820	-3.7581800	-0.6359980
 H	-3.6951160	-4.0122520	 0.2409930
 H	-2.2339760	-3.5159450	 1.1295850
 O	 4.6849630	 0.2272710	-1.4206510
 O	 3.9545410	-1.7934220	-0.6985990
 C	-1.4347430	 3.5666230	-0.4391950
 H	-2.0622450	 4.4511130	-0.4925780
 H	-0.8378680	 3.5854340	 0.4733900
 H	-0.7752310	 3.5275750	-1.3071790
 O	-2.3205330	 2.4564140	-0.4342830
 N	 2.0477800	 0.7492430	 2.2727390
 O	 1.8195320	-0.4142560	 1.8684190
 O	 2.0414050	 1.6602810	 1.4838650

TS C12
 C	 3.7919730	-0.5510910	-0.9252720
 C	-0.0540840	-0.4051660	-0.2619980
 C	-0.4391800	 0.9423240	-0.2835600
 C	-1.0392610	-1.3959370	-0.1553130
 C	-1.7825400	 1.2640640	-0.2212560
 H	 0.3198620	 1.7077990	-0.3171470
 C	-2.3865500	-1.0625740	-0.0975820
 H	-0.7208620	-2.4265530	-0.1199430
 C	-2.7666230	 0.2784490	-0.1363810
 H	 1.6085000	-1.8152530	-0.0832030
 C	 1.3382610	-0.7945720	-0.3208740
 C	 2.3851620	 0.0405510	-0.6634640
 H	 2.1936160	 0.9897790	-1.1451730
 O	-3.4052400	-1.9606830	 0.0017500
 O	-4.0820380	 0.6205470	-0.0743090
 H	-4.1144790	 1.5829230	-0.0882700
 C	-3.0463870	-3.3259880	 0.0877230
 H	-2.5190300	-3.6510020	-0.8116190
 H	-3.9800170	-3.8726780	 0.1808710
 H	-2.4217220	-3.5125420	 0.9636890
 O	 4.4914040	 0.1574420	-1.6699190
 O	 4.0141820	-1.6485530	-0.3877230
 C	-1.3483200	 3.6031080	-0.2508570
 H	-1.9288750	 4.5202570	-0.2253590
 H	-0.6891800	 3.5553000	 0.6168530
 H	-0.7544200	 3.5665250	-1.1651030
 O	-2.2891180	 2.5411450	-0.2229250
 N	 2.4700900	 0.6504870	 2.1068750
 O	 1.9543710	-0.4282520	 2.2005270
 O	 2.7915960	 1.1237330	 1.0050080

Transiton States of SA- scavenging ·OH
TS O15h
 C	-4.6818550	-0.3276910	 0.1490760
 C	-0.7741970	-0.2794240	-0.0421280
 C	-0.3397200	 1.0547460	-0.0465120
 C	 0.1740080	-1.3031870	-0.1550360
 C	 1.0050500	 1.3621530	-0.1658090
 H	-1.0733990	 1.8363910	 0.0640970
 C	 1.5172860	-0.9946560	-0.2968500
 H	-0.1670370	-2.3274420	-0.1456500
 C	 1.9524140	 0.3351800	-0.3064340
 H	-2.4142700	-1.6969350	 0.2235910
 C	-2.1856900	-0.6457240	 0.0785760
 C	-3.2374300	 0.1733410	 0.0029040
 H	-3.1347580	 1.2400230	-0.1667130
 O	 2.5251350	-1.9002490	-0.4215350
 O	 3.2549570	 0.6347990	-0.4366180
 H	 3.8148080	-0.1317750	-0.1638240
 C	 2.2097930	-3.2598680	-0.1776880
 H	 1.7595340	-3.3772360	 0.8090700
 H	 3.1521760	-3.7965540	-0.2212720
 H	 1.5303640	-3.6428690	-0.9409610
 O	-5.5333820	 0.5847560	 0.1062240
 O	-4.8269470	-1.5569650	 0.2922820
 C	 0.5871770	 3.6857970	-0.0298830
 H	 1.1786250	 4.5962560	-0.0463280
 H	-0.1181660	 3.6888720	-0.8629690
 H	 0.0384740	 3.6196930	 0.9114820
 O	 1.5129390	 2.6216900	-0.1542630
 O	 4.7198060	-0.3107550	 1.2334320
 H	 4.8525700	 0.6013560	 1.5220030

TS C11h
 C	 4.0995130	-0.3158890	-0.4672990
 C	 0.2446760	-0.3081630	 0.1110500
 C	-0.2146050	 1.0042580	 0.0254970
 C	-0.6654440	-1.3640030	 0.0944530
 C	-1.5745110	 1.2365970	-0.0985870
 H	 0.5049620	 1.8024210	 0.1204270
 C	-2.0310560	-1.1211010	-0.0361030
 H	-0.2882740	-2.3724500	 0.1718190
 C	-2.4916330	 0.1882970	-0.1382870
 H	 1.9982610	-1.4322210	 0.8018370
 C	 1.6912290	-0.5729040	 0.2190000
 C	 2.6202680	 0.0283200	-0.5695740
 H	 2.3272080	 0.7877720	-1.2842570
 O	-2.9914230	-2.0900500	-0.0695540
 O	-3.8278580	 0.4417830	-0.2517710
 H	-3.9188500	 1.3998990	-0.2743160
 C	-2.5535270	-3.4272550	 0.0554580
 H	-1.8883250	-3.7027520	-0.7659210
 H	-3.4505130	-4.0388750	 0.0184920
 H	-2.0407850	-3.5861750	 1.0066480
 O	 4.7319830	-0.2470440	-1.5338690
 O	 4.4933350	-0.6260970	 0.6842540
 C	-1.2954550	 3.5979710	-0.0465900
 H	-1.9322810	 4.4768220	-0.0873250
 H	-0.7614930	 3.5666770	 0.9038350
 H	-0.5771580	 3.6224550	-0.8672980
 O	-2.1592350	 2.4797510	-0.1713600
 O	 2.2413240	 0.7225930	 1.7966150
 H	 3.1455220	 0.3521710	 1.7274370

TS C12h
 C	 4.0538690	-0.6681260	-0.2725500
 C	 0.1717480	-0.4637910	 0.0374300
 C	-0.1523210	 0.8939100	-0.0466650
 C	-0.8521250	-1.4164080	 0.0912770
 C	-1.4837840	 1.2655490	-0.1035020
 H	 0.6503790	 1.6140690	 0.0068810
 C	-2.1866570	-1.0304860	 0.0229060
 H	-0.5809250	-2.4583390	 0.1696530
 C	-2.5092540	 0.3211690	-0.0795300
 H	 1.7970660	-1.8385970	 0.5458690
 C	 1.5656250	-0.8853430	 0.0845580
 C	 2.6090630	-0.1627950	-0.4007640
 H	 2.4382860	 0.6781740	-1.0592920
 O	-3.2461870	-1.8902040	 0.0545390
 O	-3.8133080	 0.7163490	-0.1292170
 H	-3.8008280	 1.6786970	-0.1618870
 C	-2.9465780	-3.2654580	 0.1758740
 H	-2.3528640	-3.6153700	-0.6715700
 H	-3.9034890	-3.7792120	 0.1875410
 H	-2.4091170	-3.4709400	 1.1042790
 O	 4.2189330	-1.8949000	-0.3362980
 O	 4.8957470	 0.2482770	-0.1049510
 C	-0.9404310	 3.5785190	-0.1268120
 H	-1.4757760	 4.5229730	-0.1531610
 H	-0.3483150	 3.5067200	 0.7859320
 H	-0.2807670	 3.4996790	-0.9918110
 O	-1.9333760	 2.5633940	-0.1619540
 O	 2.7076610	 1.4273700	 1.0655670
 H	 3.6556510	 1.2994240	 0.8427590

Products of SA- scavenging ·NO2 in water
P O15
 C  -4.2342280  -0.2565300 -0.0030060
 C  -0.3640230  -0.2822280  0.0052820
 C   0.0697770   1.0651070  0.0067310
 C   0.5674890  -1.3417980  0.0006090
 C   1.4070570   1.3507770  0.0042750
 H  -0.6559260   1.8623470  0.0138240
 C   1.9141980  -1.0784190 -0.0055560
 H   0.1977510  -2.3562840  0.0005540
 C   2.4136880   0.2912080 -0.0032680
 H  -1.9839410  -1.7003690  0.0394660
 C  -1.7704320  -0.6383650  0.0128330
 C  -2.8065580   0.2148140 -0.0121860
 H  -2.6702130   1.2874240 -0.0480450
 O   2.8869030  -2.0005040 -0.0130980
 O   3.6311450   0.5449790 -0.0068290
 C   2.4904060  -3.3792920 -0.0192550
 H   1.9172760  -3.6083640  0.8780030
 H   3.4136710  -3.9478030 -0.0285790
 H   1.9060880  -3.5970010 -0.9120930
 O  -5.1096270   0.6464020 -0.0995410
 O  -4.4670120  -1.4883920  0.1006370
 C   1.0350550   3.6957720  0.0221580
 H   1.6616170   4.5809940  0.0264450
 H   0.4113730   3.6828380 -0.8707220
 H   0.4190080   3.6688090  0.9200540
 O   1.9396800   2.5826580  0.0100060

P C11
 C   3.2580770   0.4134140 -1.0890310
 C   0.1818300  -0.4809980 -0.1457160
 C   0.1462650   0.8676410  0.1857440
 C  -0.9871820  -1.1805930 -0.4335010
 C  -1.0818860   1.5179680  0.2288140
 H   1.0575230   1.3983310  0.4192040
 C  -2.2135850  -0.5266380 -0.3707460
 H  -0.9317210  -2.2262680 -0.6985050
 C  -2.2603410   0.8270570 -0.0427950
 H   1.5476560  -1.7050540 -1.2174950
 C   1.4963330  -1.2385750 -0.2338090
 C   2.7374760  -0.4519190 -0.0187840
 H   3.2842080  -0.5212040  0.9085210
 O  -3.4198280  -1.1109350 -0.6192610
 O  -3.4744490   1.4572000  0.0058820
 H  -3.3251740   2.3810900  0.2467360
 C  -3.4134550  -2.5052500 -0.9428230
 H  -2.8591120  -2.6813540 -1.8641180
 H  -4.4542940  -2.7785080 -1.0809550
 H  -2.9826580  -3.0831010 -0.1256470
 O   4.2812900   1.1062610 -0.8155710
 O   2.6546050   0.4121190 -2.2006290
 C  -0.0924690   3.6015880  0.8500140
 H  -0.4468610   4.6039830  1.0652390
 H   0.4083170   3.1875180  1.7243010
 H   0.5859330   3.6221300 -0.0022820
 O  -1.2617190   2.8359370  0.5365670
 N   1.3234760  -2.2861700  1.9620320
 O   1.4693170  -2.4349270  0.6248110
 O   1.2789700  -1.1727230  2.3390220 

P C12
 C   3.7035320  -0.3785000 -0.8800930
 C  -0.1114700  -0.3778270 -0.2270910
 C  -0.5237920   0.9780280 -0.2447160
 C  -1.0924970  -1.3949480 -0.1035350
 C  -1.8671990   1.2824190 -0.1532370
 H   0.2055410   1.7683660 -0.3270360
 C  -2.4318510  -1.0705740 -0.0104360
 H  -0.7687900  -2.4248470 -0.0871010
 C  -2.8289900   0.2715580 -0.0358440
 H   1.4930940  -1.8036830 -0.3021410
 C   1.2429760  -0.7542230 -0.3282790
 C   2.3534950   0.2253480 -0.4413370
 H   2.1206770   1.0043780 -1.1687240
 O  -3.4480460  -1.9723140  0.1108420
 O  -4.1557030   0.5722670  0.0652090
 H  -4.2520380   1.5341810  0.0505940
 C  -3.0825640  -3.3542920  0.1759950
 H  -2.5798840  -3.6598070 -0.7413190
 H  -4.0143240  -3.8991740  0.2856750
 H  -2.4404730  -3.5378570  1.0369620
 O   4.7465720   0.1116410 -0.3976130
 O   3.6229890  -1.2774300 -1.7488510
 C  -1.4735750   3.6365660 -0.2556630
 H  -2.0818800   4.5347870 -0.2380560
 H  -0.7913630   3.6320860  0.5937030
 H  -0.9165730   3.5834420 -1.1903750
 O  -2.3925700   2.5424030 -0.1615850
 N   2.8321980   0.4057580  1.9171080
 O   2.9769770  -0.7609210  1.8165180
 O   2.5088100   1.0401640  0.7771630 

Products of SA- scavenging ·OH in water
P O15h
 C  -4.2342280  -0.2565300 -0.0030060
 C  -0.3640230  -0.2822280  0.0052820
 C   0.0697770   1.0651070  0.0067310
 C   0.5674890  -1.3417980  0.0006090
 C   1.4070570   1.3507770  0.0042750
 H  -0.6559260   1.8623470  0.0138240
 C   1.9141980  -1.0784190 -0.0055560
 H   0.1977510  -2.3562840  0.0005540
 C   2.4136880   0.2912080 -0.0032680
 H  -1.9839410  -1.7003690  0.0394660
 C  -1.7704320  -0.6383650  0.0128330
 C  -2.8065580   0.2148140 -0.0121860
 H  -2.6702130   1.2874240 -0.0480450
 O   2.8869030  -2.0005040 -0.0130980
 O   3.6311450   0.5449790 -0.0068290
 C   2.4904060  -3.3792920 -0.0192550
 H   1.9172760  -3.6083640  0.8780030
 H   3.4136710  -3.9478030 -0.0285790
 H   1.9060880  -3.5970010 -0.9120930
 O  -5.1096270   0.6464020 -0.0995410
 O  -4.4670120  -1.4883920  0.1006370
 C   1.0350550   3.6957720  0.0221580
 H   1.6616170   4.5809940  0.0264450
 H   0.4113730   3.6828380 -0.8707220
 H   0.4190080   3.6688090  0.9200540
 O   1.9396800   2.5826580  0.0100060

P C11h
 C   3.4544210  -0.3708840 -0.6775160
 C   0.1864200  -0.6938480  0.4020970
 C   0.2242360   0.6990440  0.3557380
 C  -1.0000010  -1.3849310  0.1863960
 C  -0.9487060   1.3958430  0.1007930
 H   1.1569830   1.2186140  0.5202900
 C  -2.1732550  -0.6771900 -0.0752690
 H  -1.0030520  -2.4642920  0.2286520
 C  -2.1466310   0.7132970 -0.1152740
 H   1.2135540  -2.5143630  0.7827360
 C   1.4772810  -1.4631300  0.6565750
 C   2.3470120  -1.3256020 -0.5516440
 H   2.0702480  -1.8930280 -1.4285510
 O  -3.3921330  -1.2485820 -0.2984760
 O  -3.3077250   1.3954090 -0.3710240
 H  -3.1079630   2.3408690 -0.3654120
 C  -3.4518580  -2.6783030 -0.2798110
 H  -2.8061100  -3.0977930 -1.0508530
 H  -4.4863680  -2.9312720 -0.4878050
 H  -3.1664830  -3.0593910  0.7002820
 O   4.0419060  -0.3005490 -1.7915820
 O   3.7645720   0.3330890  0.3417230
 C   0.1464310   3.5180090  0.1517750
 H  -0.1466900   4.5549430  0.0270900
 H   0.5882070   3.3721310  1.1368380
 H   0.8530780   3.2331950 -0.6269960
 O  -1.0596910   2.7552500  0.0284790
 O   2.1217250  -1.0317480  1.8513150
 H   2.7932650  -0.3763900  1.5570780

P C12h
 C  -4.0122330  -0.4404450  0.3503850
 C  -0.2142950  -0.3837470 -0.0161740
 C   0.1982580   0.9700270  0.0607500
 C   0.7730100  -1.4010470 -0.0700270
 C   1.5452240   1.2718610  0.0915570
 H  -0.5354130   1.7600550  0.0881490
 C   2.1159640  -1.0791750 -0.0358650
 H   0.4520260  -2.4298880 -0.1338120
 C   2.5120520   0.2606240  0.0480850
 H  -1.8222200  -1.7974900 -0.1371710
 C  -1.5758180  -0.7479310 -0.0452600
 C  -2.6924940   0.2454530 -0.0303290
 H  -2.5107400   1.0157920  0.7229990
 O   3.1376490  -1.9830860 -0.0793130
 O   3.8441680   0.5592540  0.0807510
 H   3.9382660   1.5193240  0.1440380
 C   2.7763680  -3.3633730 -0.1845990
 H   2.1947490  -3.6731980  0.6833970
 H   3.7128630  -3.9105510 -0.2155090
 H   2.2129110  -3.5417110 -1.1000120
 O  -4.0536730  -1.0009800  1.4698720
 O  -4.9409870  -0.3793020 -0.4935860
 C   1.1476350   3.6255980  0.2015510
 H   1.7560890   4.5223680  0.2540890
 H   0.5413960   3.6405380 -0.7035450
 H   0.5120710   3.5544410  1.0835520
 O   2.0698950   2.5308820  0.1640860
 O  -2.8284680   0.8915070 -1.3056610
 H  -3.7226810   0.6470510 -1.5958030   
